# Artificial Intelligence Predictions in Huge Chemical Spaces: Chiroptical Properties of [6]‐helicene Family

**DOI:** 10.1002/advs.74715

**Published:** 2026-03-13

**Authors:** Rafael G. Uceda, Sandra Míguez‐Lago, Carlos M. Cruz, Boris Pérez‐Cañedo, Alfonso Gijón, Luis Álvarez de Cienfuegos, Antonio J. Mota, Delia Miguel, Juan M. Cuerva

**Affiliations:** ^1^ Departamento de Química Orgánica Facultad De Ciencias Universidad De Granada (UGR) Granada Spain; ^2^ Departamento de Matemáticas Universidad De Córdoba (UCO) Córdoba Spain; ^3^ Instituto De Investigación Biosanitaria Granada Spain; ^4^ Departamento De Química Inorgánica UEQ UGR Facultad De Ciencias Granada Spain; ^5^ Departamento De Fisicoquímica UEQ UGR Facultad De Farmacia Granada Spain; ^6^ Unidad de Excelencia de Química Aplicada a la Biomedicina y Medioambiente (UEQ) Universidad de Granada Granada Spain

**Keywords:** [6]helicenes, artificial intelligence, chiroptical properties, computational chemistry, helical structures

## Abstract

Navigating the vast chemical space remains a major challenge in the rational design of materials with tailored properties. Here, we investigate how the properties of the [6]helicene family can be effectively modelled using a local, data‐driven AI framework. By predicting each molecule from its closest structural neighbours, we accurately estimate diverse photophysical and (chir)optical properties. The coupling with genetic algorithms enables efficient inverse design and multi‐objective optimization, yielding molecules unlikely to arise from intuition alone. The method uncovers [6]helicenes with enhanced electronic circular dichroism (ECD) features, tuned low‐energy transitions, and exceptionally large *g* values, while revealing clear structure–property relationships that translate into practical design rules. Overall, this framework offers a general and efficient route for goal‐directed molecular discovery across extensive chemical spaces.

## Graphical abstract

This study shows that a local, data‐driven AI model can accurately predict diverse optical and chiroptical properties of [6]helicenes using information from close structural neighbours. Combined with genetic algorithms, it enables inverse design for tailored properties, establishing practical structure–property rules for efficient molecular discovery.

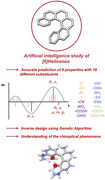

## 1 Introduction

One of the main aims in organic chemistry is the search of molecules with desired properties. Nevertheless, it is worth noting that, considering the current organic chemistry knowledge, an astonishing number (10^33^ to 10^60^) of synthesizable organic molecules can now be prepared [1, 2, 3]. The question is how to search for promising organic structures in a systematic way, considering such a vast chemical space. Obviously, the costly and time‐consuming synthesis and analysis of all possible molecules is not an efficient approach, and the available routine computational methods for estimating ground‐state photophysical properties cannot handle such a vast number of structures. The challenge is even greater for excited‐state properties, since more accurate and computationally demanding calculations are required [4, 5]. In contrast, machine learning (ML) techniques have emerged as powerful tools to address these kind of problems [6, 7, 8, 9, 10, 11, 12]. Ideally, one would have a priori a complete dataset containing all the relevant properties for every molecule—an unrealistic but conceptually perfect scenario. In such a case, experimental efforts in the laboratory could be minimized, as the properties of the target molecules would already be known. In reality, any available dataset will inevitably be incomplete. Yet, having a fully exhaustive dataset might not be strictly necessary if the existing data efficiently and uniformly cover the chemical space. In this scenario, the properties of the molecules in the empty regions of the dataset could, in principle, be interpolated or predicted based on the existing data. Within this context, although optimal, the idea of a “universal” predictor is rather naïve, as molecular properties are complex phenomena that depend on multiple and diverse factors. However, when these properties vary smoothly across structurally similar molecules, a more practical solution becomes feasible. In such cases, an ad hoc predictor for any given property could be developed using only data from a local neighbourhood, thereby decomposing the global complexity into manageable sub‐problems with different underlying factors. Although appealing, the success of this approach ultimately relies on the quality of the database and the presence of an underlying physical consistency within the localized dataset.

**FIGURE 1:**
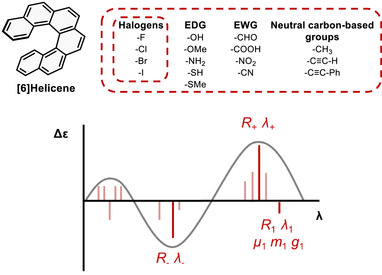
[6]Helicene and the chemical groups (up) and optical and chiroptical properties (down) studied in this work.

We recently described that Neural Networks (NN), trained with DFT‐generated data, were able to simulate one chiroptical property (maximum rotatory strength *R*) of (poly)halogenated [6]helicenes, a family covering a chemical space of ∼7 × 10^10^ compounds [13]. Here, we communicate that the locally driven approach introduced here, where each compound is predicted based on its neighbours, is more general and has been now successfully applied to a much more complex problem: the evaluation of different chiroptical properties of prototypical [6]helicenes. This new space is composed of [6]helicene derivatives substituted by halogens (−F, −Cl, −Br, −I), electron donor groups (EDG: ─NH_2_, ─OH, ─OMe, ─SH, ─SMe), electron acceptor groups (EWG: ─NO_2_, ─CN, ─CHO, ─COOH), carbon‐based groups (─CH_3_, ─C≡CH, ─C≡C─Ph) and their combinations, thus covering different electronic characteristics (Figure 1, upper part). The training dataset included [6]helicenes with up to six substituents, a limit that can be easily increased on demand. Moreover, that number was also chosen based on practical considerations, as synthesizable molecules hardly contain more substituents and compounds with a large number of substituents may be less appealing to experimentalists. Consequently, the following discussions refer to results mainly restricted to mono‐ to hexasubstituted [6]helicenes. This work represents a proof of concept of the viability of the local approach, providing accurate tendencies and good predictions, and covering a huge chemical space of around 7 × 10^10^ compounds. Higher substitutions can be also studied simply adding the corresponding data to the dataset.

As targeted properties, we have selected nine key descriptors: i) the maximum value of positive (*R_+_*) and negative rotatory strengths (*R–*) with their corresponding wavelengths (*λ_+_*, *λ–*), which define the main profile of an electronic circular dichroism (ECD) spectrum, ii) the rotatory strength of the least energetic transition (*R_1_*) and its wavelength (*λ_1_*), iii) the corresponding electric and magnetic dipole transition moments for the S_0_→S_1_ transition (*µ* and *m*, respectively) and iv) its corresponding absorption dissymmetry factor, *g_abs_* value (Figure 1, lower part). The latter value describes the unbalanced absorption of circularly polarized photons by chiral entities in the ground state, where *g_abs_* = ±2 for pure left/right circularly polarized absorbing transitions, whereas rather low values in the range of *g_abs_* = 10^−5^ are expected for poorly dissymmetric responses. For isotropic samples, it can be described in terms of two vectors, *g*_abs_ ≈ 4│*m*││*µ*│ cos*θ*/(│*µ*│^2^ +│*m*│^2^)), being │*m*│ and │*µ*│ the modules of the electric and magnetic dipole transition moments and *θ* the angle between the corresponding vectors for the S_0_→S_n_ transitions [14, 15, 16]. Additionally, emission dissymmetry factors (*g_lum_*) can be inferred for rigid structures from the *g*_abs_ values of the lowest‐energy transition, since it is assumed that structural relaxation of the excited state is minimal [17, 18]. Consequently, the *g* value of the S_0_→S_1_ transitions are expected to be closely related to those of the S_1_→S_0_ transitions. These values lie at the core of circularly polarized luminescence (CPL) [19, 20], a property considered essential for developing emerging smart technologies [21, 22, 23]. In this context, the applicability and predictive capability of the present model will be evaluated through a series of representative case studies.

## 2 Results and Discussion

### 2.1 Data Set and Model Training

A predictive model must be trained using the most diverse and accurate data available [24, 25, 26, 27]. Accordingly, the dataset should be randomly selected to include molecules that cover a broad and representative portion of the chemical space, ensuring diverse structural and property values [28]. In our case, the use of DFT‐calculated data guaranties their homogeneity and the possibility of generating more examples to cover other regions of the chemical space if needed.

In this case, the dataset comprises more than 13 000 (*P*)‐[6]helicenes containing up to six substituents. These data are organized into six families: four individual families, each corresponding to a specific type of substituent; a fifth family combining one substituent of each group (─F, ─CN, ─OMe, and─C≡CH), which serves to evaluate the transferability and generalization of the patterns learned from the individual cases; and a sixth family that includes all 16 possible substituents in various combinations.

For each compound, we have considered the following properties, extracted from DFT calculations: *R_+_*, *R–, R*_1_, *λ_+_*, *λ–*, *λ_1_*, │*m*│, │*µ*│, and *g_abs_*. It is worth noting that the calculations were made with the *P*‐enantiomer. For chiroptical predictions, the conclusions of this study can be applied to the opposite *M* helical configuration by simply inverting the sign for chiroptical parameters. All the (chir)optical properties were calculated using DFT methods at the M06 [29]/TZVP [30] level of theory as implemented in Gaussian 09 (see SI) [31].

**FIGURE 2:**
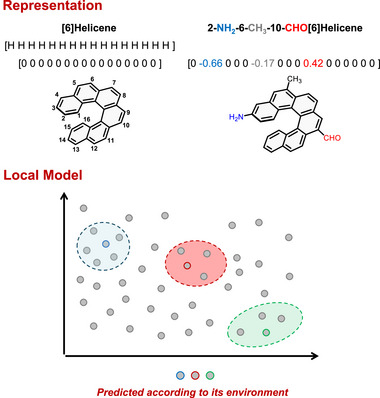
Representation of the [6]helicenes using a 1D vector and Hammett´s sigma constant (up) and representation of the local model strategy (down).

The success of the local model relies on a molecular representation in which the similarities and the physics of the system can be properly represented [32, 33]. Considering that all the input molecules share the same carbo[6]helicene skeleton, we decided to represent the helicene as a vector constituted by 16 elements representing the substitution of the molecule (Figure 2, upper part). In our previous work, simple labels described the nature of the substituents, however, herein we have represented them by their electronic nature, described by the *para* Hammett´s sigma (σ*_p_*) [34]. This parameter acts as a chemically meaningful descriptor that captures electronic effects and provides the model with the required information to perform accurate property regressions.

Beyond dataset quality and representation, model performance depends on how the local neighbourhood is defined. Here, each molecule´s neighborhood consists of the “n” closest molecules according to the weighted Euclidean distance (see SI for details). Testing different neighbourhood sizes, show that one hundred neighbours gave the best regression performance across all molecules and properties, although an additional fine‐tuning can be performed for specific cases when required.

Once the local neighbourhood is defined, the regression problem is established. Although representing samples as pairs ⟨X, Y⟩, where X denotes the molecular representation and Y the property to be predicted, yielded good results with Neural Networks (NN), locally trained Random Forest (RF) models provided even better performance metrics (Figures S1 and S2):This suggests that our dataset is sufficiently rich for new molecules to find similar examples from which to learn. The use of locally trained RF models significantly reduces the computational cost associated with training a global model for the whole dataset. The integration of the local approach with RF offers a methodologically simple yet conceptually robust framework, enabling efficient adaptation to diverse chemical environments, as only structurally and electronically similar molecules contribute to each localized model, while dissimilar cases are automatically disregarded (Figure 2, lower part). It is important to note that the objective here is not to achieve a perfect quantitative description of the systems, but rather to identify candidate molecules with the desired properties and general trends. Consequently, the success of the model should be evaluated not only in terms of conventional performance metrics, but also by its effectiveness in proposing suitable candidates.

### 2.2 Model Performance

**FIGURE 3:**
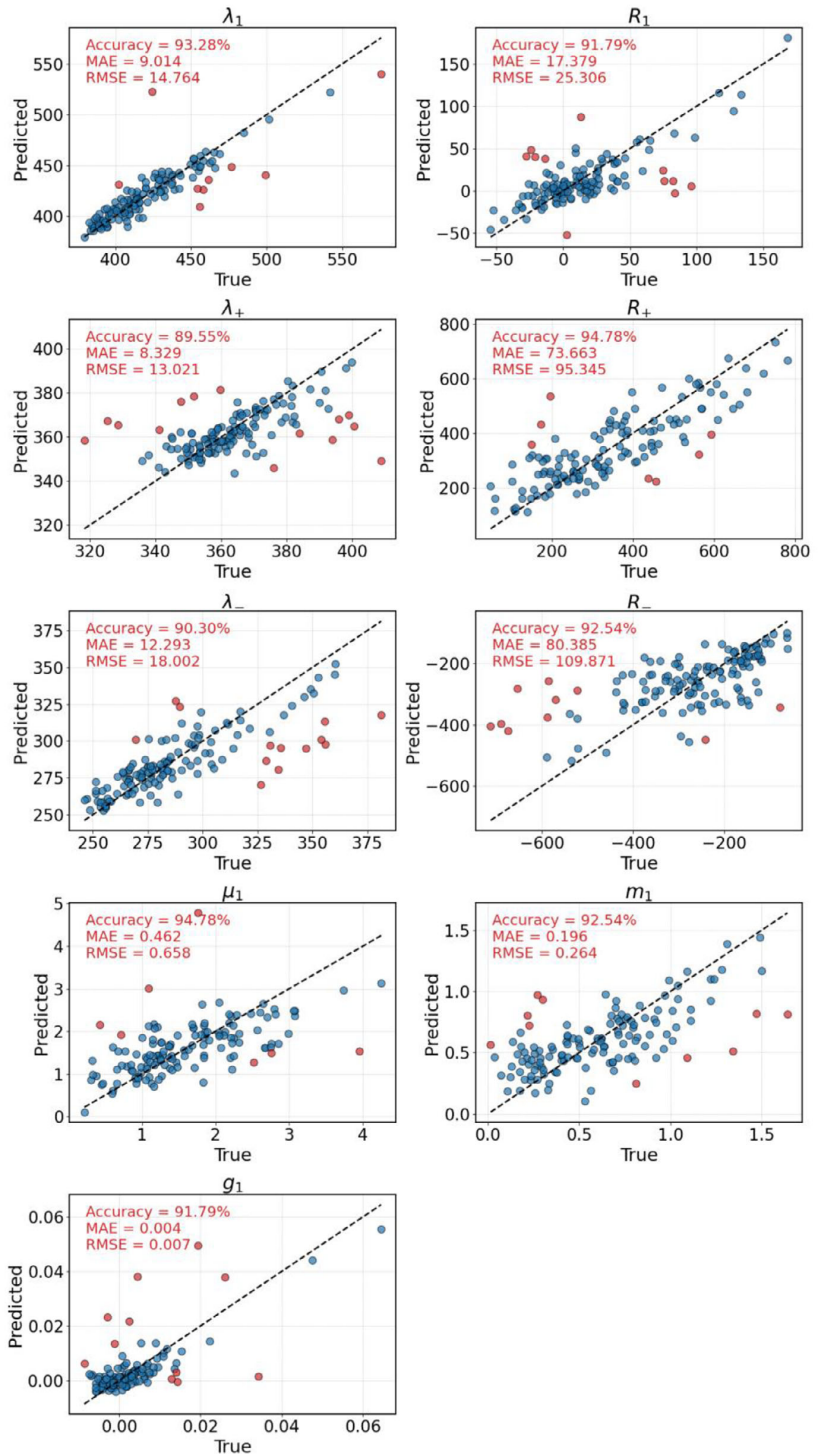
Regression of the target properties using the local RF model, evaluating only 1% of the total dataset for visualization purposes. Blue points exhibit an error below 2.5 median absolute deviations, while red points show an error above that threshold. The Mean Absolute Error (MAE) and Root Mean Square Error (RMSE) metrics are computed over the entire set of points.

With the developed model in hand, its performance was evaluated using a randomly selected 20% subset of data (see SI for details). For each tested compound, a local model was constructed using the 100 most similar training neighbours. Across all investigated properties, the mean absolute error (MAE) remains within a reasonable range, with the majority of the predicted values closely following the diagonal in the corresponding parity plots. Although a few outliers are observed—defined as data points deviating more than 2.5 times the scaled median absolute deviation from the expected trend—they typically represent less than 10% of the total dataset (Figure 3).

Particularly noteworthy is the performance observed for *g*_abs_, one of the key magnitudes of chiroptical properties. It is important to note that, when using a global model based on all available data points, the results for *g*_abs_ were significantly less informative (Table S2 and Figures S1 and S2), highlighting the necessity of a more refined approximation strategy — namely the local approach. The localized framework enables the capture of finer structural and property‐dependent variations that are not well represented in a purely global model, thereby enhancing both the robustness and predictive reliability of the methodology.

### 2.3 Optimization Algorithms

The main goal of our models is to design new compounds with the desired properties in a rational way. A possible approach is to run the model and exhaustively evaluate millions or billions of candidate molecules, and subsequently select those that exhibit the target properties. However, this brute‐force strategy quickly becomes infeasible in contexts such as ours, where the number of possible compounds reaches 10^11^. A more efficient and attractive alternative involves coupling our local predictive models with optimization algorithms to directly design compounds that achieve a specific target property values. For this purpose, we used genetic algorithms (GAs) [35, 36, 37], which are particularly useful in problems where the search space is extremely large or no simple a priori path exists to explore it, as frequently occurs in chemistry and molecular design.

These algorithms mimic the principles of natural selection, iteratively generating and refining candidate molecules to effectively navigate complex chemical spaces. The following cases will illustrate the capability of this approach to optimize several properties simultaneously, demonstrating the versatility and power of our integrated predictive‐optimization framework.

It is worth noting at this point that the cases presented here are independent examples; however, as will be shown later, they can be combined, and our algorithms are capable of handling multiple variables and constraints simultaneously. Thus, the traditional direct strategy, guided by chemical intuition, consist of synthesizing molecules and then determine the chiroptical properties. With the proposed combination of local models and genetic algorithms, an **inverse design**, where the value of the desired property is known before the synthesis, can be developed. Regarding the presentation of results, the molecules are named using the Position‐Substituent scheme (e.g., 1‐SH_3‐F_8‐OH), allowing immediate identification of substituent count and placement.

### 2.4 Inverse Design: Maximizing R_+_

**FIGURE 4:**
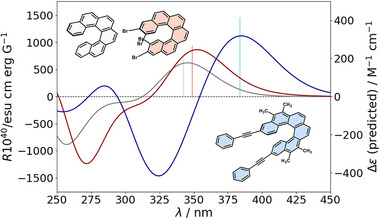
Predicted transitions (bars) and ECD spectra (continuum) of the proposed candidates to enhance the rotatory strength *R*_+_.

In our previous work, statistical analysis revealed that the chiroptical response of a halo[6]helicene is limited to a maximum *R_+_* value of approximately 1150 (in 10^−40^ esu cm erg G^−1^) and among the studied derivatives, the highest response was observed for 2‐Br_3‐Br_14‐Br_15‐Br, with 942 (Figure 4, red). This observation prompts a key question: can the combined incorporation of the new substituents lead to an enhanced chiroptical response exceeding this limit? Guided by these considerations, the GA was executed with an initial population of 1000 individuals to ensure adequate exploration of the chemical space and sufficient representation of the sparsely populated regions in the original dataset (see SI). As a result, several promising candidates bearing alkynes were identified (Table S3), whose properties were subsequently validated through DFT calculations, notably highlighting compounds with values up to 1486 (Figure 4, blue). This finding is consistent with concepts previously developed by our research group [38, 39, 40, 41, 42, 43, 44, 45, 46], as the presence of alkynes enhances │*m*│ and, ultimately, the rotatory strength (*R*) of the transition.

The GA can also be configured to exclude alkynes from the search, enabling the exploration of alternative chemical spaces. Under these constraints, multiple candidates were identified with *R_+_* values around 850, generally featuring bromine or iodine at positions 2 and 3 (Table S3), in agreement with our previous results. This is particularly interesting because they are consistent with those reported in our earlier study, despite using a completely different approach. These results demonstrate how a rapid evaluation strategy, which does not require millions of individual calculations, can effectively maximize key chemical properties.

### 2.5 Inverse Design: Simultaneous Maximization of R_+_ and R–

**FIGURE 5:**
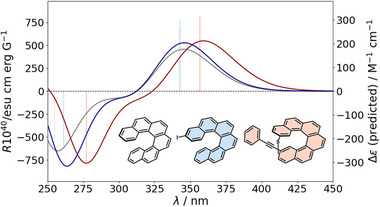
Predicted transitions (bars) and ECD spectra (continuum) of the proposed candidates to simultaneously enhance *R*_+_ and *R–*.

Having validated our approach for a single property, we next addressed the simultaneous optimization of two variables, aiming, for instance, to identify systems in which both *R*_+_ and *R–* exceed the values observed for the parent [6]helicene (694 and –514, respectively). The GA again showed high effectiveness, yielding multiple candidates with values surpassing those of the pristine [6]helicene (Table S4). Interestingly, all of them share a low degree of substitution, reinforcing our previous conclusions that increased substitution tends to localize molecular orbitals, reduce *m*, and consequently diminish the chiroptical response. Notable candidates proposed by the algorithms and validated by DFT calculations were 2‐SMe_15‐C≡CH (*R*_+_ = 818, *R–* = −764, Figure 5, red), 3‐CN_14‐COOH (*R*_+_ = 753, *R–* = −686) or 3‐COOH_13‐F_14‐CN (*R*_+_ = 763, *R–* = −686). It is also worth noting that very simple compounds such as 3‐I[6]helicene (*R*_+_ = 778, *R–* = –615, Figure 5, blue) and 3‐Cl[6]helicene (*R*_+_ = 713, *R–* = –566), also satisfy the criteria and confirm our previous observation that substitution at position 3 is unique for the enhancement of both *R*_+_ and *R–*. Its identification by the GA not only validates these theoretical insights but also demonstrates that the model can autonomously capture the underlying patterns and optimize multiple chiroptical properties simultaneously.

### 2.6 Inverse Design: Simultaneous Maximization of R_+_/R– and λ_+_/λ–

After maximizing chiroptical responses, another promising idea consists in the simultaneous optimization of *R* and the excitation energy of the process (*λ*). While the previous sections focused on enhancing only the magnitude of the chiroptical response for applications such as sensing [47], in many practical scenarios it is also desirable to shift the response towards lower energies [48, 49]. Under this premise, we executed the GA with the goal of simultaneously obtaining large values for both *R*_+_ and *λ*_+_, with the challenge of surpassing pristine [6]helicene (694 and 342.6 nm, respectively). Acceptable candidates were readily identified (Table S5), among the selected molecules we can highlight 2‐CN_3‐COOH_10‐SMe_14‐C≡CH_15‐C≡C‐Ph (*R*_+_ = 898, *λ*_+_ = 391.2) and 2‐CN_3‐COOH_10‐CH_3__14‐C≡CH_15‐C≡CH_16‐F (*R*_+_ = 759, *λ*_+_ = 374.1).

The same protocol was applied to the *R–*/*λ–* pair (−514 and 254 nm for parent [6]helicene) and among the solutions (Table S6), notable examples include 3‐CHO_9‐COOH_11‐COOH_14‐CHO (*R–* = −708, *λ−* = 284.3) and 5‐F_14‐SH (*R−* = −780, *λ−* = 268.0). It is important to note that this scenario does not address the absolute maximum absorption wavelength of [6]helicenes, but rather the simultaneous optimization of two magnitudes associated with the *B*_b_ and *B*_a_ bands [50, 51], revealing the versatility of the inverse design approach in capturing complex multi‐objective relationships.

### 2.7 Inverse Design: Maximization of M_1_

**FIGURE 6:**
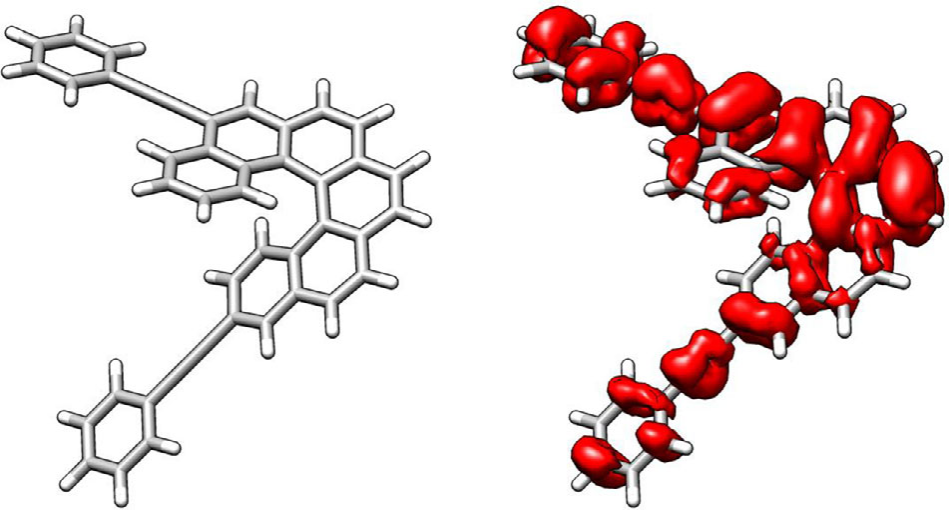
Left: Chemical structure of 5‐C≡C─Ph_14‐C≡C─Ph (*m* = 3.51). Right: *m* density (isosurface: 0.05) for the S_0_→S_1_ transition.

Having analysed the parameters of the main bands in the ECD spectrum, we next focused in the lowest energy electronic transition, involved in different and significant processes. Initially, its *m* –namely *m_1_*‐ can be considered, being a key quantity in chiroptical properties [52]. Furthermore, it has previously been proposed by our group as a predictive parameter for spin filtering [44]. With this insight, the design of molecules exhibiting large *m*_1_ values becomes particularly interesting for numerous applications of the CISS effect [53, 54]. The GA revealed clear preferences for certain positions and substituents to enhance this magnitude. Positions 2, 3, and 5 appear optimal whereas candidates rarely feature substitutions at positions 6, 7, and 8. Regarding the substituents, a marked preference was observed for alkynes, bromine, iodine, and functional groups such as ─NH_2_ or ─SMe (Table S7). The latter are particularly noteworthy, as they can serve as anchoring groups in various experimental setups, such as unimolecular conductance measurements in STM‐BJ experiments [55, 56]. Taken together, these studies provide clear design rules, with predicted *m*_1_ values reaching up to 3 (in × 10^−20 ^erg G^−1^), significantly surpassing the value of 0.36 observed for pristine [6]helicene. In particular, the largest example identified is 5‐C≡C‐Ph_14‐C≡C─Ph (*m*_1_ = 3.51, Figure 6), where analysis of the density using MultiWFN software [57, 58] reveals that the conjugation of the alkynes extends the *π*‐system, spreading the *m* density across the entire helical structure.

### 2.8 Inverse Design: Design Compounds With a Preferred λ_1_

After the lowest energy transition, we next focused on the transition energy. An especially interesting aspect in this context is not merely maximizing or minimizing a property, but rather designing systems to attain a specific target value. Focusing on the transition wavelength, we first asked the GA to identify candidates with *λ*_1_ ≈ 400 nm, a region of the property distribution with a high density of examples. The algorithm successfully retrieved suitable molecules (Table S8), including some bearing up to seven substituents, demonstrating its capability to explore and generalize within well‐sampled regions. Encouraged by these results, we extended the analysis to additional targets of 450 nm (Table S9) and 500 nm (Table S10). In all cases the algorithm efficiently located promising candidates exhibiting transition energies remarkably close to the desired values, such as 2‐SH_3‐C≡CH_8‐Br_9‐NH_2__14‐C≡CH_15‐C≡CH (*λ*_1_ = 451.39 nm) or 1‐NO_2__3‐NO_2_ (*λ*_1_ = 500.66 nm). Importantly, the core idea here is not merely to find compounds with absorption at 400, 450, or 500 nm, but rather to demonstrate the capability to design molecules with tailored properties—a principle that can be extended to multiple contexts. Moreover, the resulting structures often display non‐obvious substitution patterns, which represents an additional strength of the model, as such molecules would likely remain undiscovered through conventional approaches.

**FIGURE 7:**
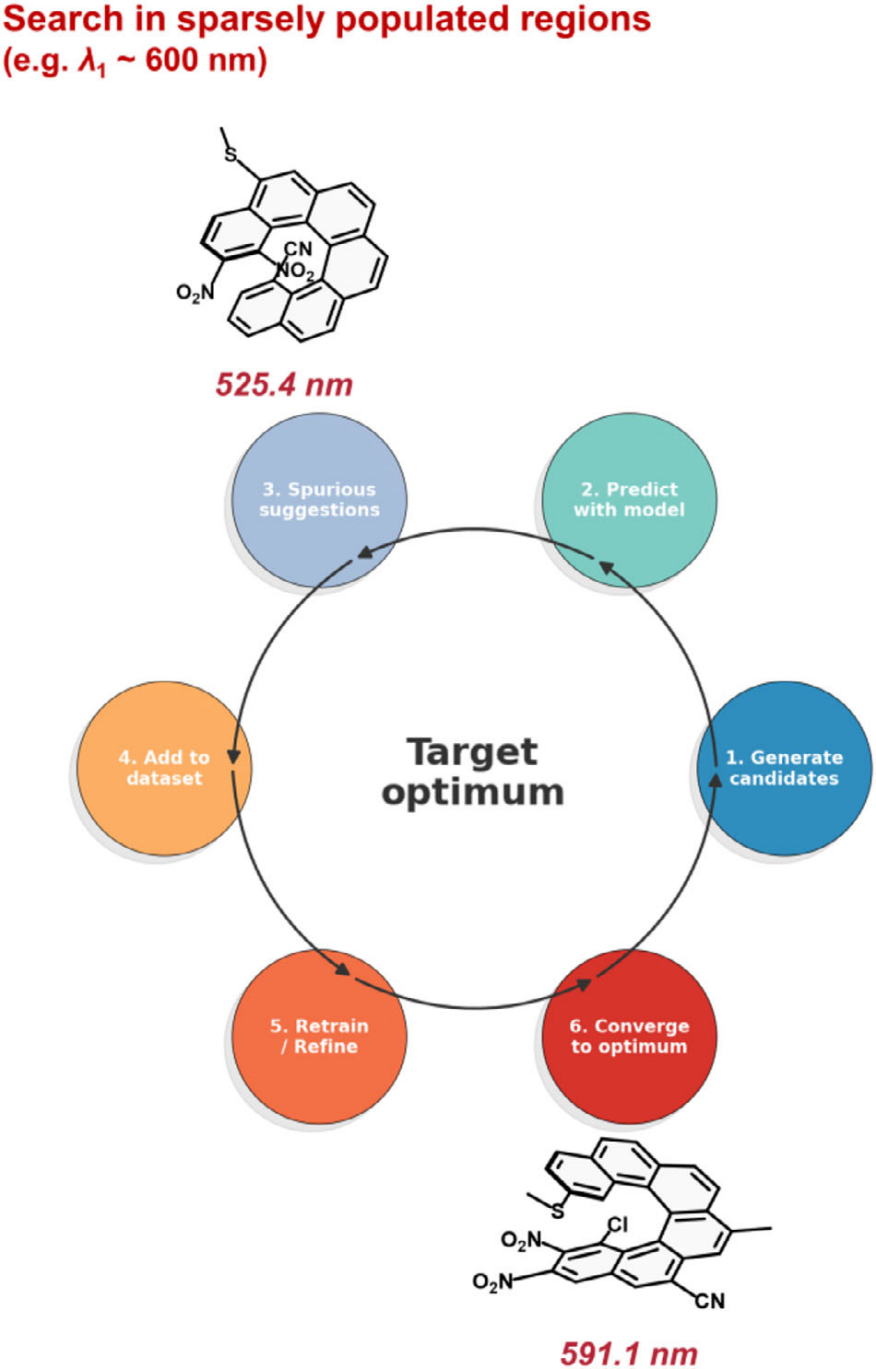
Scheme of the iterative protocol.

Building on this, we next explored the scarce regions of the property distribution, targeting transitions at 550 and 600 nm. In these cases, the first iteration of the genetic algorithm produced candidates with *λ*_1_ values far from the intended targets (e.g., around 520 nm for the 600 nm case, Figure 7). However, this is precisely where another strength of our methodology becomes evident: by including the intermediate candidates in the dataset and iteratively retraining and reapplying the algorithm, the search gradually converged toward the desired region (Figure 7), ultimately identifying compounds with *λ*_1_ = 551.8 and 598.8 nm, respectively (Tables S11 and S12).

These results demonstrate that the proposed framework not only enables the precise tuning of properties in well‐sampled regions but also allows the progressive exploration and optimization of scarcely populated areas of chemical space, providing a powerful route toward a truly goal‐directed molecular design.

### 2.9 Exceptional g_abs_ Values for the Less Energetic Transition in *P*‐[6]helicenes

The dissymmetry factor *g_abs_* is one of the key parameters in chiroptical response and it can be equally studied using our local RF+GA combination. The purpose of including *g_abs_* in this study is to identify molecules presenting exceptional values, far beyond the typical range of 10^−3^‐10^−4^, a usual behaviour that arises from the disparity of three orders of magnitude between *m* and *µ*. Despite the structural complexity of the molecules, the only relevant contributions come from the orbitals involved in the transition and while chemical intuition can help to design structures with reasonably high *g_abs_* values, realistic estimation of hidden parameters such as *µ*, *m*, and cos *θ* is beyond human capabilities.

The S_0_→S_1_ transition is particularly important because it is directly related to an emerging property: CPL. The *g_abs_* value for this transition determines the maximum efficiency of the corresponding CPL response. As stated, the physical limit of *g_abs_* is 2; however, the maximum value found in simple organic molecules using rational design of the transition moments is only 0.2 [59] and the examples beyond 0.05 are already exceptional [60, 61, 62, 63, 64, 65, 66].

A natural question arises: what is the maximum *g_abs_* value achievable across the entire family of (*P)*‐[6]helicenes? To address this, we turn to GAs and optimization techniques. Even with these powerful tools, finding high *g_abs_* values is far from trivial: values above 0.01 are already extraordinary [67, 68, 69], and the search can easily become trapped in local maxima. Thus, identifying candidates with *g_abs_* approaching or exceeding 0.1 is extremely rare and underscores the difficulty of approaching the theoretical limit of 2 in a challenging context.

After running the GA with several modifications (see SI for details), we identified multiple candidates exceeding this threshold of 0.1 (Table S13). Notably, none of these molecules could have been conceived a priori through chemical intuition. Instead, they were uncovered within an enormous and virtually incommensurable chemical space, far beyond the scope of conventional rational design. DFT calculations confirmed that the predicted values closely match the algorithmic estimations. Among these candidates, several helicenes exhibited remarkable *g_abs_* values, with 1‐OH_3‐SH_6‐SH standing out as the most significant example, displaying an extraordinary *g_abs_* of 0.389—an exceptional value both for a helicene and for a simple organic molecule.

The underlying reason for these outstanding responses lies in their remarkably small *µ* (∼0.01 in × 10^−18^ esu cm, compared with a conventional value of approximately (1), which greatly amplifies the magnetic‐to‐electric dipole ratio that defines *g*_abs_. In other words, these molecules achieve a strong chiroptical response not through an unusually large *m*, but rather through a highly suppressed electric counterpart—a delicate balance that is exceedingly rare and difficult to predict through traditional design approaches. Although these compounds may possess thermally accessible conformers with higher electric dipole moments, which could lead to a reduction in the dissymmetry factor *g*, the present discussion focuses on a conceptual point: the identification of intrinsically high dissymmetry values at the level of the energy minima, beyond practical conformational effects. In any case, for the compounds exhibiting the highest *g*_abs_ values, the different conformers were explicitly analyzed, revealing a consistent behavior across all cases (Table S14). Overall, this example underscores the conceptual and methodological strength of our approach, demonstrating its ability to identify extraordinarily rare and exceptional candidates within a chemical space containing billions of possibilities.

### 2.10 Exceptional CPL Emitters in (*P*)‐[6]helicenes

Perfect CPL emitters are unknown to date and represent a challenge from a fundamental point of view. Nevertheless, the previous section is dedicated to *g_abs_*, and a close relationship exists between such values and *g_lum_* [70]. Consequently, high *g_abs_* values are related with high *g_lum_* values. Nevertheless, a key difference exists: CPL depends on the emissive properties of the material, which is related with *µ*. While an optimized CPL emitter should ideally exhibit a large *g_lum_*, this condition alone is insufficient, since transitions with very low oscillator strength *f* (i.e., small │*µ*│) may lead to non‐emissive states [71]. This is precisely the scenario discussed in the previous section, where │*µ*│ was well below the threshold for emissivity. Therefore, │*µ*│ must exceed a certain threshold—approximately 1—to ensure observable emission, but, it should not be excessively large, as a partially “forbidden” (low │*µ*│) transition is still required to maintain high dissymmetry. High *m* combined with an almost perfect alignment between *µ* and *m* (cos *θ* ≈ 1) can “close the loop”, yielding *g_lum_* values far beyond the typical 10^−3^ limit.

Following this rationale, the goal of this section is to identify privileged emitters in which both *g_lum_* and the transition moments are simultaneously optimized. Using the same GA framework, numerous candidates satisfying both requirements were identified, many of them sharing common substitution patterns—most notably the presence of a NO_2_ group at position 1 (Table S15). Representative examples include 1‐NO_2__16‐CN (*g_abs_* = 5.08 10^−2^, │*µ*│ = 0.93) and 1‐NO_2__4‐OH_12‐F (*g_abs_* = 5.6 10^−2^, │*µ*│ = 0.84). This behaviour can be attributed to a synergistic interplay between both vectors: the NO_2_ substituent at position 1 slightly increases │*m*│ while reducing │*µ*│ just enough to enhance the dissymmetry without fully suppressing emission (see Tables S16 and S17).

To further probe whether other substitution patterns could achieve similar performance, additional GA runs were conducted under the constraint of excluding NO_2_ groups. Interestingly, new candidates featured alternative electron‐withdrawing substituents at the terminal positions of the helicene core (Table S18). Notable examples include 1‐CHO_15‐CHO (*g_abs_* = 1.49 10^−2^, │*µ*│ = 3.9) and 2‐C≡CH_10‐CN_13‐COOH_14‐NH_2__16‐CN (*g_abs_* = 1.1 10^−2^, │*µ*│ = 3.58).

Finally, since all these predictions were based on the S_0_→S_1_ transitions, corresponding emission processes were subsequently computed using TD‐DFT for the selected compounds, revealing consistent *g*_lum_ values—many of them well above the 10–^2^ threshold. This approach is particularly noteworthy, as it represents the first artificial intelligence‐driven study explicitly addressing the transition moments governing CPL emission, marking a significant step toward data‐guided molecular photonic design.

### 2.11 Exceptional CPL Emitters in (*P*)‐[6]helicenes: What About Their Synthesis?

The main aim despite scientific curiosity is the preparation of the target molecules. Although the model can propose interesting candidates, the reality is that many of them face complex or long synthetic routes. At this stage, it is worth noting that additional constraints can be imposed on the genetic algorithm to guide the search toward the most synthetically accessible molecules. For example, the algorithm can be configured to generate molecules with a specified number of substituents, predefined functional groups or to avoid chemically incompatible combinations.

**FIGURE 8:**
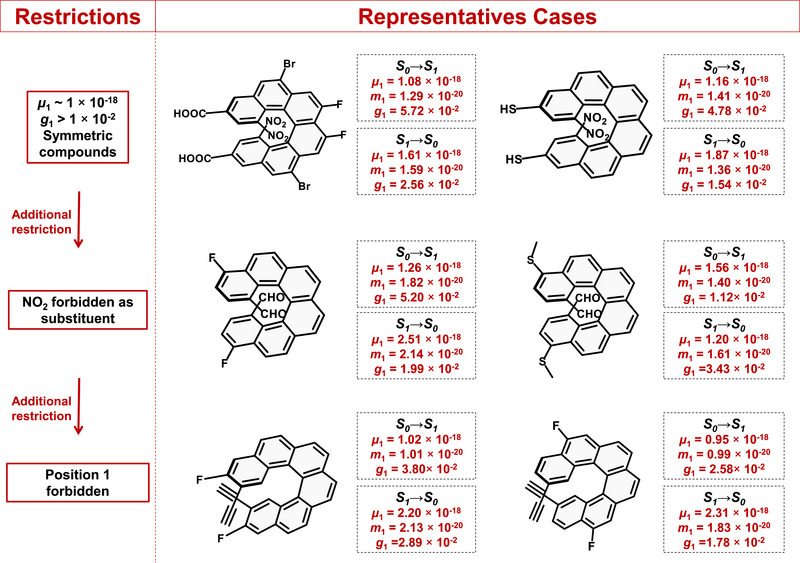
Design of exceptional CPL emitters under different restrictions.

As a first illustration, symmetry constraints were imposed on the candidate molecules, facilitating their synthesis. The genetic algorithm was then run under three constraints (symmetric molecules, *µ ∼* 1, *g_abs_* > 1 × 10^−2^, Figure 8, upper part), yielding multiple derivatives, once again highlighting the presence of NO_2_ at positions 1 and 16 (Table S19). The procedure was subsequently repeated while prohibiting NO_2_, introducing a fourth constraint (Figure 8, center), which also generated multiple viable candidates (Table S20). Finally, recognizing that position 1 (or 16, by symmetry) is typically substituted, we imposed an additional restriction forbidding its use (Figure 8, lower part). This resulted in a genetic algorithm run with five distinct constraints, encompassing property requirements (*µ* and *g_abs_*), positional restrictions (position 1 prohibited), chemical logic (NO_2_ forbidden), and structural considerations (molecular symmetry). Under these conditions, the algorithm typically produced helicenes featuring alkynes at position 2 (Table S21), while maintaining dissymmetry factors on the order of 10^−2^ for both the S_0_→S_1_ and S_1_→S_0_ processes.

It is worth noting that, in this final scenario, the dissymmetry factors are somewhat lower, which is entirely reasonable, since any molecules outperforming the original designs would likely have been selected in earlier runs rather than those featuring NO_2_ at position 1. Figure 8 summarizes the various adjustments implemented in this study, illustrating how the methodology can be easily customized to limit the number of substituents, the type of functional groups, or the positions involved, while still guiding the search toward synthetically feasible [6]helicenes.

### 2.12 Opening the Black Boxes: From AI Predictions to Molecular Design

Thus far, we have built predictive models for vast [6]helicene chemical spaces, complemented by genetic algorithms that guide the design toward desired property profiles. Additionally, we have demonstrated that these approaches can indeed identify high‐performing candidates; however, the next step is to determine which specific structural combinations give rise to the desired properties. This shift transforms the models from mere prediction engines into tools for extracting chemically interpretable rules. In line with the previous sections, we now focus on exceptional CPL emitters, where gaining this level of clarity allows us to open two “black boxes” at once: that of the AI models—where interpretability is a persistent challenge—and that of chiroptical properties, traditionally understood only through laborious trial‐and‐error exploration.

**FIGURE 9:**
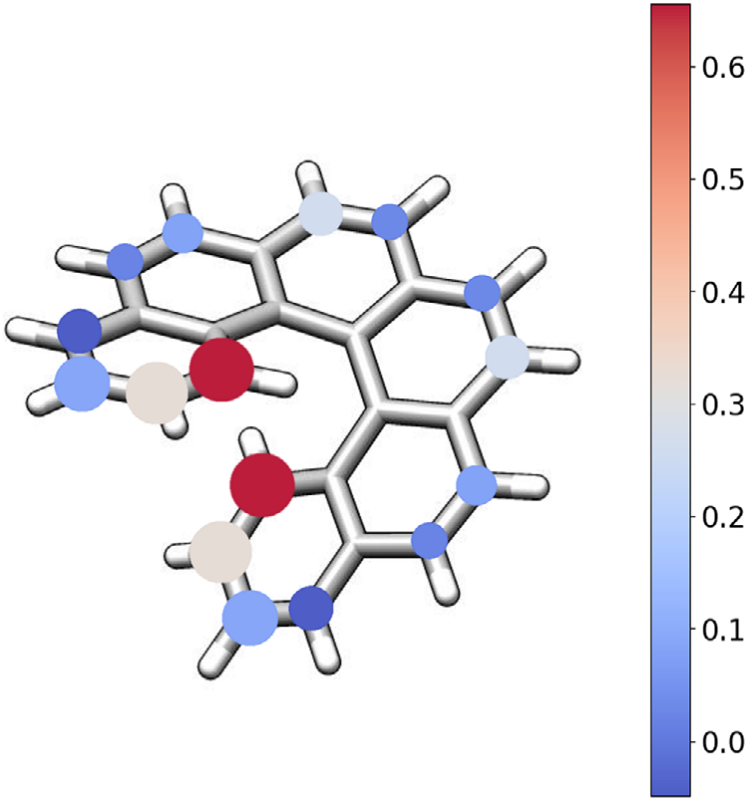
Scheme of the substitution patterns in exceptional emitters.

Using the data obtained in the previous section, we analysed substituents frequency and positions (Figure 9), where circles size reflects how frequently that site is substituted, and the colour encodes the average Hammett constant at that position. Clear patterns emerge and position 1 is typically substituted with strongly electron‐withdrawing groups, while position 2 is also frequently modified, typically with substituents exhibiting Hammett constants around 0.30. The remaining positions show much lower substitution and their average Hammett values are near to zero, indicating a marked preference for retaining the original hydrogen atoms.

Guided by these straightforward yet informative trends, we imagined a series of tetrasubstituted [6]helicenes incorporating –CN or –NO_2_ at Position 1 and ─Cl, –Br, –C≡CH, or –C≡C–Ph at position 2. The resulting eight compounds were evaluated via TD‐DFT, and the absorption and emission calculations reveal a consistent picture: most structures exhibit *g* values on the order of 10–^2^ and electric dipole transition moments in the emissive range (Table S22). For example, we can highlight 1‐NO_2__2‐Br_15‐Br_16‐NO_2_ (*g_abs_* = 5.18 × 10^−2^, │*µ*│ = 0.94) or 1‐CN_2‐C≡CH_15‐C≡CH_16‐CN (*g_abs_* = 3.38 × 10^−2^, │*µ*│ = 1.71). These results demonstrate how patterns extracted from (“black box”) models can be directly translated into rational molecular design, effectively bridging the gap between prediction and understanding. Thus, we are not only identifying excellent emitters, but also understanding exceptional molecular families, creating a synergy between artificial intelligence and chemical intuition.

## 3 Conclusions

This study demonstrates the power of combining local artificial intelligence models with genetic algorithms for the rational design of [6]helicenes across a huge chemical space of ∼7 × 10^10^ compounds, enabling accurate predictions of complex chiroptical properties. This results in an efficient inverse design, which can be used to identify molecules with specific target values for a property, enabling a sophisticated and precise molecular design. When applied to the dissymmetry factor, *g*_abs_, the method successfully maximizes it, reaching values on the order of 10^−1^ for simple organic molecules. Many of these high‐performing structures feature non‐intuitive substitution patterns that would likely remain undiscovered using traditional trial‐and‐error approaches, underscoring the strength of a data‐driven, algorithmically guided strategy. Nevertheless, the process can be customized according to user requirements, specifying the number of substituents, the type of functional groups, or the positions to be modified. Importantly, the protocol described here can be readily adapted to other families of compounds or to optimize different molecular properties, demonstrating its broad applicability and potential to transform artificial intelligence predictions into actionable chemical intuition across diverse chemical domains.

## Conflicts of Interest

The authors declare no conflicts of interest.

## Supporting information




**Supporting File**: advs74715‐sup‐0001‐SuppMat.pdf.

## Data Availability

The data that support the findings of this study are available in the supplementary material of this article.
