# Supplementary material for: Artificial Intelligence Predictions in Huge Chemical Spaces: Chiroptical Properties of [6]‐helicene Family
Source: Adv Sci (Weinh). 2026 Mar 13;13(28):e74715. doi: 10.1002/advs.74715 (PMC13185875; doi:10.1002/advs.74715)
Supplement: Supplementary file 1 — Supporting File: advs74715‐sup‐0001‐SuppMat.pdf. [file ADVS-13-e74715-s001.pdf]

Supporting Information
©Wiley-VCH 2026
69451 Weinheim, Germany

Artificial Intelligence predictions in huge chemical spaces: chiroptical properties of [6]-helicene family

Rafael G. Uceda,[a] Sandra Míguez-Lago,[a] Carlos M. Cruz,[a] Boris Pérez-Cañedo,[a] Alfonso Gijón,[b] Luis Álvarez de Cienfuegos,[a][c] Antonio Mota,[d]* Delia Miguel,[e]* and Juan M. Cuerva[a]*

[a] R. G. Uceda, Dr. S. Míguez-Lago, Dr. C. M. Cruz, Dr. B. Pérez-Cañedo, Prof. L. Álvarez de Cienfuegos, Prof. J. M. Cuerva
Departamento de Química Orgánica, Unidad de Excelencia de Química Aplicada a la Biomedicina y Medioambiente (UEQ)
Universidad de Granada (UGR), Facultad de Ciencias
C. U. Fuentenueva, 18071 Granada, Spain
E-mail, jmcuerva@ugr.es

[b] Dr. A. Gijón
Departamento de Matemáticas
Universidad de Córdoba (UCO)
C.U. Rabanales 14014 Córdoba (Spain)

[c] Prof. L. Álvarez de Cienfuegos
Instituto de Investigación Biosanitaria.
Avda. Madrid, 15, 18016 Granada (Spain).

[d] Dr. A. J. Mota
Departamento de Química Inorgánica, UEQ, UGR
Facultad de Ciencias, C. U. Fuentenueva, 18071 Granada, Spain
E-mail, mota@ugr.es

[e] Dr. D. Miguel
Departamento de Fisicoquímica, UEQ, UGR
Facultad de Farmacia, Avda. Profesor Clavera s/n
C. U. Cartuja, 18071 Granada, Spain
E-mail, dmalvarez@ugr.es

Abstract: Navigating the vast chemical space remains a major challenge for the rational design of materials with tailored properties. Here, we investigate the [6]helicene family—a prototypical class of chiral polycyclic aromatics—and show that their properties can be effectively modelled using a local, data-driven AI framework. By predicting each molecule from its closest structural neighbours, this approach accurately estimates diverse (chir)optical properties, including rotatory strengths, transition wavelengths, dipole transition moments, and dissymmetry factors. Coupled with genetic algorithms, it enables efficient inverse design and multi-objective optimization, yielding molecules unlikely to arise from intuition alone. The method uncovers [6]helicenes with enhanced ECD features, tuned low-energy transitions, and exceptionally large *g* values, while revealing clear structure–property relationships that translate into practical design rules. Overall, this framework offers a general and efficient route for goal-directed molecular discovery across extensive chemical spaces.

Table of Contents

1. Dataset generation. ... 3
2. Local model details. ... 5
3. Local model performance. ... 6
4. Genetic algorithms details. ... 10
5. Optimization cases: detailed results ... 11
5.1. Maximizing R_+ ... 11
5.2. Maximizing R_+ and R_- ... 12
5.3. Maximizing R_+ and λ_+ ... 13
5.4. Maximizing R_- and λ_- ... 13
5.5. Maximizing m_1 ... 14
5.6. Designing λ_1 ... 15
5.6.1. λ_1 ~ 400 nm ... 15
5.6.2. λ_1 ~ 450 nm ... 16
5.6.3. λ_1 ~ 500 nm ... 17
5.6.4. λ_1 ~ 550 nm ... 18
5.6.5. λ_1 ~ 600 nm ... 18
5.7. Exceptional g_{abs} values for the less energetic transition in *P*-[6]helicenes ... 19
5.8. Exceptional CPL emitters in (*P*)-[6]helicenes ... 20
5.9. Exceptional CPL emitters in (*P*)-[6]helicenes: what about their synthesis? ... 23
5.10. Opening the black boxes: from AI Predictions to Molecular Design ... 26
6. References. ... 27

1. Dataset generation

[6]Helicene derivatives were generated using a Python-based automated procedure employing random number selection to identify the positions of the helicene framework that would be substituted by groups other than hydrogen. Integers from 1 to 16 were generated, corresponding to each substitution site.

After selecting a position, a random substituent from a predefined list was assigned. This list included the following substituents: -F, -Cl, -Br, -I, -OMe, -SH, -SMe, $-NH_2$, -CHO, -COOH, $-NO_2$, -CN, $-CH_3$, -C≡CH, and -C≡C-Ph. The procedure was repeated until the desired degree of substitution was achieved, ensuring that each generated position was unique and therefore that no substituent was assigned to the same site more than once within the [6]helicene structure.

The generated [6]helicenes can be classified into six families: four comprising molecules substituted exclusively with a single type of functional group (halogens, electron-withdrawing groups, electron-donating groups, and neutral carbon-based groups), a fifth family containing one representative substituent from each category (-F, -CN, -OMe, -C≡CH), and a sixth family in which all sixteen substituents are randomly mixed. The aim of this classification was, on the one hand, to capture the characteristic chemical patterns associated with each family and, on the other, to enable the identification of combined patterns that emerge only when substituents of different chemical nature are present simultaneously. This hybrid design provides an efficient means of sampling the vast chemical space associated with the ten possible substitution patterns. As a result, and after multiple iterations aimed at enlarging the dataset to improve the statistical metrics, a final dataset comprising 13350 molecules was obtained. This total also reflects the inclusion of [6]helicenes that were not accurately computed in certain design scenarios; incorporating them here contributes to refining subsequent predictions and design strategies.

Finally, for their representation within the artificial intelligence framework, each [6]helicene is converted into a 16-element vector in which each entry corresponds to one of the sixteen substitution sites. The numerical value assigned to each position encodes the substituent present at that site, using its Hammett σ_p constant as the descriptor.[1]

Once the corresponding [6]helicenes had been generated, their structures were evaluated using DFT calculations performed with the Gaussian 09 software package.[2] All computations were carried out employing the M06 functional[3] and the TZVP basis set,[4] together with the SDD pseudopotential for iodine.[5] In addition, a Polarizable Continuum Model (PCM)[6] was applied to account for the solvent effects of dichloromethane, corresponding to the conditions under which the spectra are expected to be experimentally recorded.

In our previous study,[7] we assessed the viability of this computational approach by examining the reported spectra of pristine [6]helicene, 2-bromo[6]helicene, 2-amino[6]helicene, 2,15-dicyano[6]helicene, and 2,15-dimethoxy[6]helicene. In that work, we showed that hybrid functionals with a moderate percentage of Hartree–Fock exchange, such as B3LYP and M06, are particularly well suited for these systems, as they predict chiroptical properties with high accuracy. However, while the previous study mainly focused on the shape of the ECD spectra and the rotatory strengths, the present work additionally considers the dissymmetry factor. Consequently, an explicit discussion of the computational evaluation of g_{abs} is necessary.

Accordingly, the compounds were computed using the B3LYP,[8] M06, and PBE0[9] functionals, in all cases in combination with the TZVP basis set (Table S1). The results are very clear and display highly consistent trends across all cases. For instance, regardless of the functional employed, pristine [6]helicene systematically exhibits the lowest dissymmetry factor and magnetic dipole moment among the selected compounds. Likewise, the effect of bromination at the 2 and 3 positions is consistently observed: the introduction of bromine atoms leads to an increase in both the transition magnetic moment and the dissymmetry factor in all cases. Overall, the observed trends are clear and coherent.

We also wish to comment on the values obtained for the compounds designed according to the guidelines presented in the final section of the manuscript. Within this set, all dissymmetry factors fall in the 10^{-2} range, confirming their exceptional character irrespective of the functional employed. Taken together, these results demonstrate a remarkable robustness of both the relative rankings and, to a large extent, the absolute values of the calculated chiroptical parameters.

Table S1. Dissymmetry factors and transition dipole moments computed using various functionals.

	M06			B3LYP			PBE0		
**Compound**	**$\mu_1 10^{18}$/esu cm**	**$m_1 10^{20}$/erg G^{-1}**	**g_1**	**$\mu_1 10^{18}$/esu cm**	**$m_1 10^{20}$/erg G^{-1}**	**g_1**	**$\mu_1 10^{18}$/esu cm**	**$m_1 10^{20}$/erg G^{-1}**	**g_1**
[6]helicene	0.77	0.36	1.57×10^{-3}	0.74	0.30	1.09×10^{-3}	0.79	0.33	2.21×10^{-3}
2-bromo [6]helicene	1.04	0.66	5.30×10^{-3}	1.12	0.65	5.03×10^{-3}	1.27	0.68	5.65×10^{-3}
2,15-dibromo [6]helicene	1.32	1.01	1.10×10^{-2}	1.44	0.97	9.20×10^{-3}	1.61	1.03	1.04×10^{-2}
2,3,14,15-tetrabromo [6]helicene	1.38	1.21	1.18×10^{-2}	1.34	1.02	1.02×10^{-2}	1.45	1.03	1.19×10^{-2}
1-NO_2_2-Br_15-Br_16-NO_2 [6]helicene	0.94	1.35	5.18×10^{-2}	0.97	1.08	3.83×10^{-2}	0.68	0.94	3.82×10^{-2}
1-CN_2-Br_15-Br_16-CN [6]helicene	1.76	1.81	1.44×10^{-2}	1.92	1.63	1.35×10^{-2}	1.72	1.67	1.95×10^{-2}
1-NO_2_2-Cl_15-Cl_16-NO_2 [6]helicene	0.92	1.31	5.15×10^{-2}	0.95	1.09	3.98×10^{-2}	0.66	0.94	3.87×10^{-2}
1-CN_2-Cl_15-Cl_16-CN [6]helicene	1.82	1.67	1.00×10^{-2}	1.92	1.62	1.35×10^{-2}	1.72	1.67	1.95×10^{-2}

2. Local model details.

The ML workflow was implemented in Python and relies on *pandas*, *NumPy* and *scikit-learn* libraries. The procedure begins by defining the sixteen structural positions of the [6]helicene framework, which are encoded as the columns *Pos_1* through *Pos_16*. The dataset is then imported from a CSV file and a feature matrix is constructed by extracting only the positional descriptors.

A local prediction function was developed to estimate target properties using a hybrid nearest-neighbour and ensemble-regression strategy. For a given query molecule, the function first removes the entry corresponding to the query itself as well as its inverted analogue, ensuring that no symmetry-related duplicates bias the training subset. A *k*-nearest-neighbours model (Euclidean metric) is then applied to identify the *top_n* most structurally similar molecules. These neighbours define a local training set for a Random-Forest regressor, trained with inverse-distance weights to emphasize closer analogues. The model subsequently returns a locally informed prediction for the query vector.

Model performance is assessed through a dedicated evaluation routine. This function filters out incomplete entries and optionally selects a random fraction of the data for computational efficiency. For each molecule in this sample, leave-one-out validation is performed: the local prediction function is executed while the molecule under evaluation is temporarily removed from the training pool. The predicted and reference values are stored for later analysis.

After the full validation loop, prediction errors are computed and statistical outliers are identified as points lying outside a ±2.5×MAD interval around the mean error, where MAD is the median absolute deviation scaled to provide a robust estimate of the standard deviation. Accuracy is then defined as the percentage of data points that fall within this interval. The function also reports the mean absolute error (MAE) and the root-mean-square error (RMSE).

3. Local model performance.

To benchmark the predictive performance of the present local model, based on Nearest Neighbors selection followed by Random Forest regression (RFlocal), we compared it against the global neural network model (NNglobal) developed in a previous study,[7] which was trained on the full dataset. For this comparison, we performed a 5-fold cross-validation over a randomly selected 20% subset of the data. In the case of the local model, predictions for each molecule rely exclusively on information from chemically similar neighbors. Conversely, the global neural network is trained on the remaining 80% of each fold.

Table S2 reports the performance metrics obtained for all molecular targets. In addition to the standard MAE, RMSE, and R^2, we include the normalized metrics nMAE and nRMSE, computed by dividing the raw errors by two standard deviations of the corresponding dataset, following common normalization practices. The error bars reported in the table correspond to the standard deviation across folds. Overall, the RFlocal model consistently displays better performance across all metrics.

Table S2. Performance metrics for the predicted properties.

	**MAE**		**RMSE**		**R^2**		**nMAE**		**nRMSE**	
**Target**	**Local**	**Global**	**Local**	**Global**	**Local**	**Global**	**Local**	**Global**	**Local**	**Global**
R_1	19.0±0.4	13.3 ± 0.6	32.0±1.0	34.0 ± 4.0	0.38± 0.01	0.31 ± 0.05	0.227 ± 0.005	0.162 ± 0.009	0.39 ± 0.01	0.42 ± 0.01
R_+	76.0±1.0	84.0 ± 2.0	102.0± 2.0	146.0 ± 10.0	0.58± 0.01	0.19 ± 0.04	0.237 ± 0.004	0.26 ± 0.01	0.318 ± 0.005	0.45 ± 0.01
R_-	78.0±1.0	77.5 ± 0.6	104.0± 2.0	98.4 ± 0.8	0.37± 0.02	0.44 ± 0.02	0.294 ± 0.005	0.296 ± 0.005	0.393 ± 0.007	0.376 ± 0.006
μ_1	0.507± 0.007	0.65 ± 0.01	0.8± 0.02	0.88 ± 0.03	0.33± 0.02	0.2 ± 0.03	0.254 ± 0.003	0.33 ± 0.005	0.401 ± 0.009	0.447 ± 0.008
g_1	0.00422± 0.00005	0.0072 ± 8e-05	0.01± 0.001	0.0102 ± 0.0003	0.34± 0.08	0.2 ± 0.02	0.188 ± 0.002	0.316 ± 0.009	0.42 ± 0.07	0.448 ± 0.004
m_1	0.214± 0.004	0.158 ± 0.003	0.298± 0.006	0.36 ± 0.03	0.4± 0.01	0.14 ± 0.02	0.275 ± 0.005	0.2 ± 0.02	0.383 ± 0.008	0.463 ± 0.006
λ_1	12.8±0.1	32.1 ± 0.7	30.0± 3.0	41.9 ± 0.9	0.58± 0.07	0.15 ± 0.02	0.14 ± 0.001	0.352 ± 0.003	0.32 ± 0.03	0.46 ± 0.005
λ_+	10.25± 0.07	11.5 ± 0.1	15.3± 0.3	16.4 ± 0.2	0.32± 0.02	0.24 ± 0.02	0.273 ± 0.002	0.305 ± 0.004	0.408 ± 0.007	0.436 ± 0.007
λ_-	14.1±0.2	15.8 ± 0.6	20.9± 0.4	24.0 ± 1.0	0.42± 0.01	0.2 ± 0.03	0.253 ± 0.003	0.293 ± 0.004	0.377 ± 0.006	0.447 ± 0.008

The same results are shown graphically in Figure S1 as bar plots. The RFlocal model (blue) yields lower error metrics (MAE, RMSE, nMAE, nRMSE) and higher R^2 compared to the NNglobal model (red). To assess the statistical significance of these differences, we conducted paired t-tests between the two models for each target. Statistically significant improvements ($p < 0.05$) overwhelmingly favor the RFlocal model.

These results are further summarized in the heatmap shown in Figure S2, where the color encodes the relative performance difference (in %) between NNglobal and RFlocal. Blue regions indicate superior RFlocal performance, while white cells and asterisks denote non-significant differences between the two models. Although blue dominates across all metrics, it is particularly pronounced in the R^2 column, indicating that the local model more effectively captures global trends and data variability. While error-based metrics show relative improvements of 15–25%, the differences in R^2 reach approximately 40% in favor of the RFlocal model.

Taken together, these analyses demonstrate that the local Random Forest model provides superior predictive accuracy, especially for extrapolating to unseen molecules and guiding molecular design or property exploration.

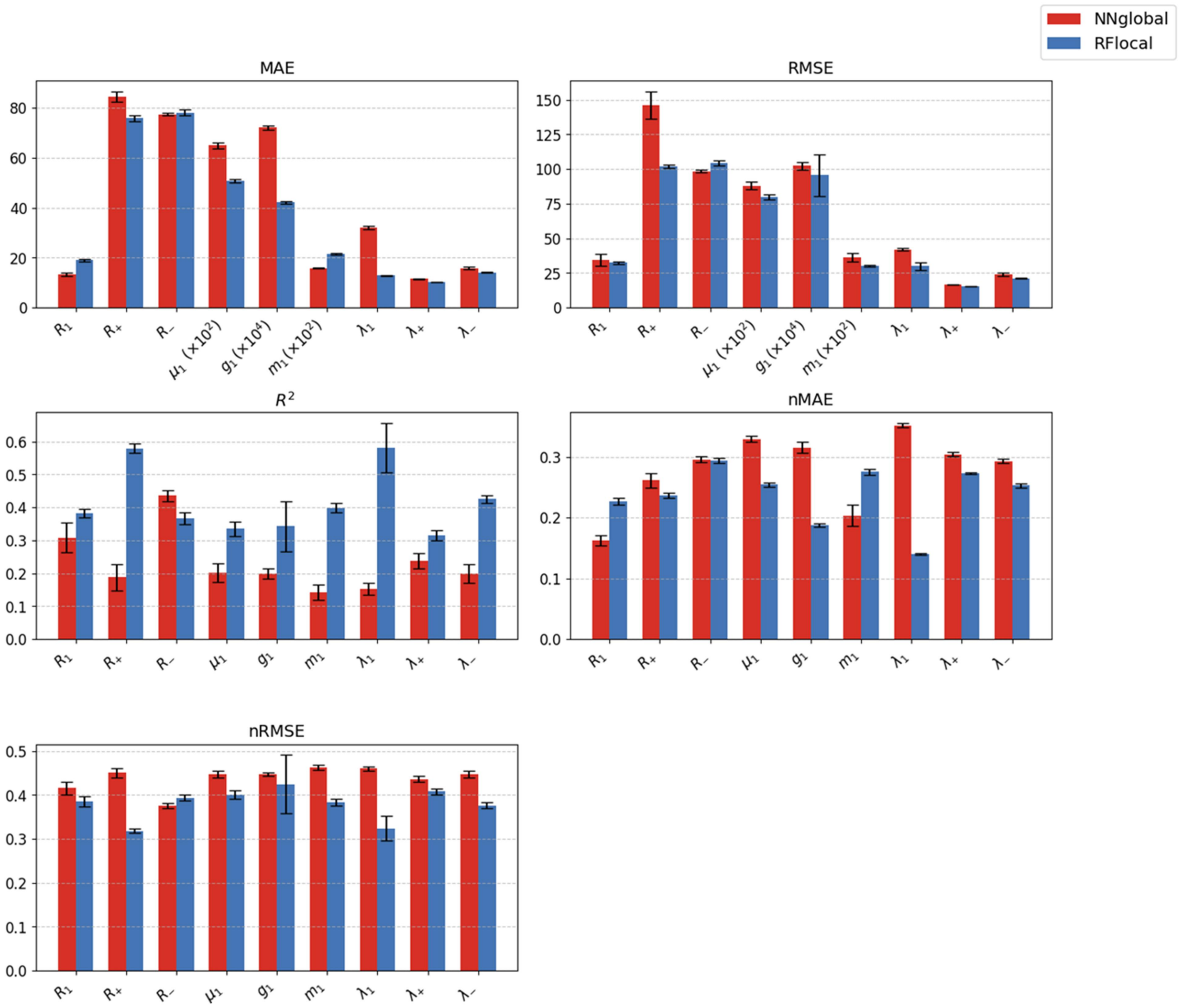

Figure S1. Comparison of predictive performance between the RFlocal and NNglobal models across all targets. Bar plots report mean values over the 5-fold cross-validation, with error bars representing the standard deviation across folds.

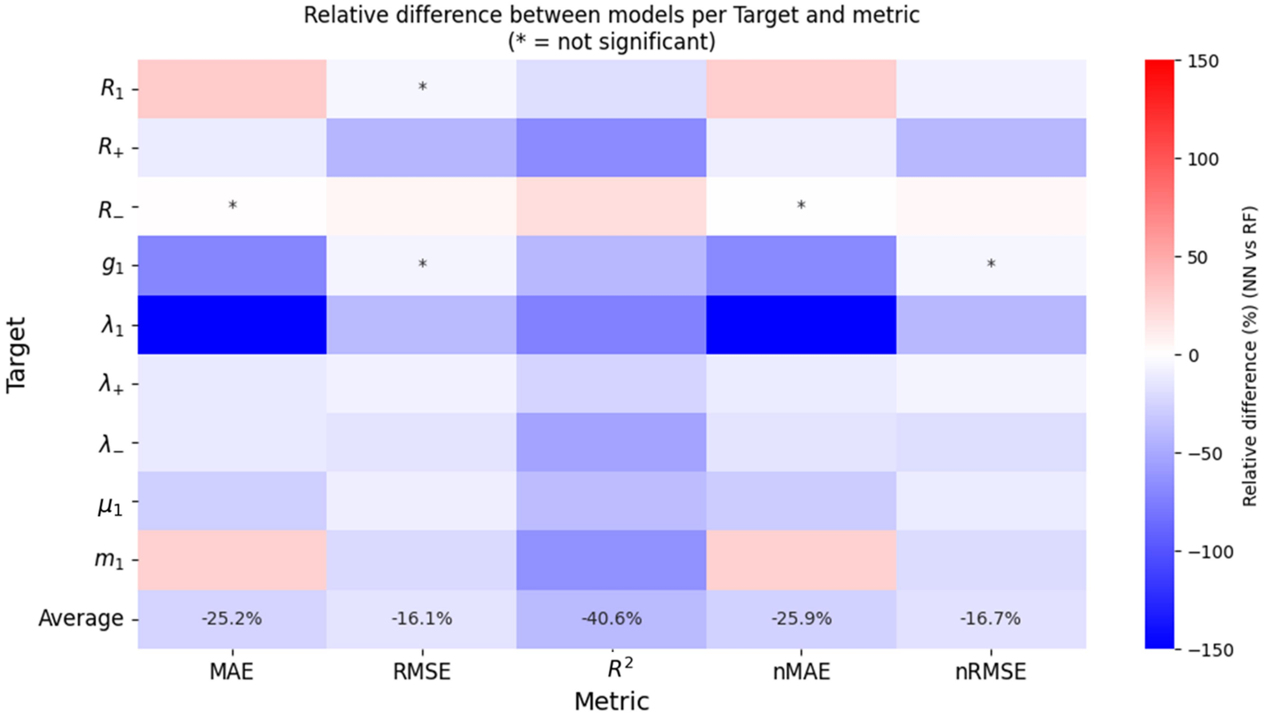

Figure S2. Heatmap of the relative differences in accuracy between the NNglobal and RFlocal models for all targets and metrics. Colors indicate the percentage relative improvement (centered at zero), and asterisks denote cases where differences are not statistically significant (paired *t*-test, $p > 0.05$).

Beyond predictive accuracy, we also analyzed the confidence and calibration of the RFlocal model. To do so, we evaluated the prediction error on a held-out 20% of the dataset. Using the mean absolute error (MAE) as an empirical estimate of σ, we quantified the predictive uncertainty by defining confidence intervals of the form $\pm k \cdot \sigma$, and computed, for each factor k, both the coverage (percentage of predictions falling within the uncertainty interval) and the mean interval width. Across all properties, a factor of 1.5·σ yields a coverage close to 80%, as shown in Figure S3, indicating that the RFlocal model provides uncertainty estimates that are both reasonably calibrated and practically informative.

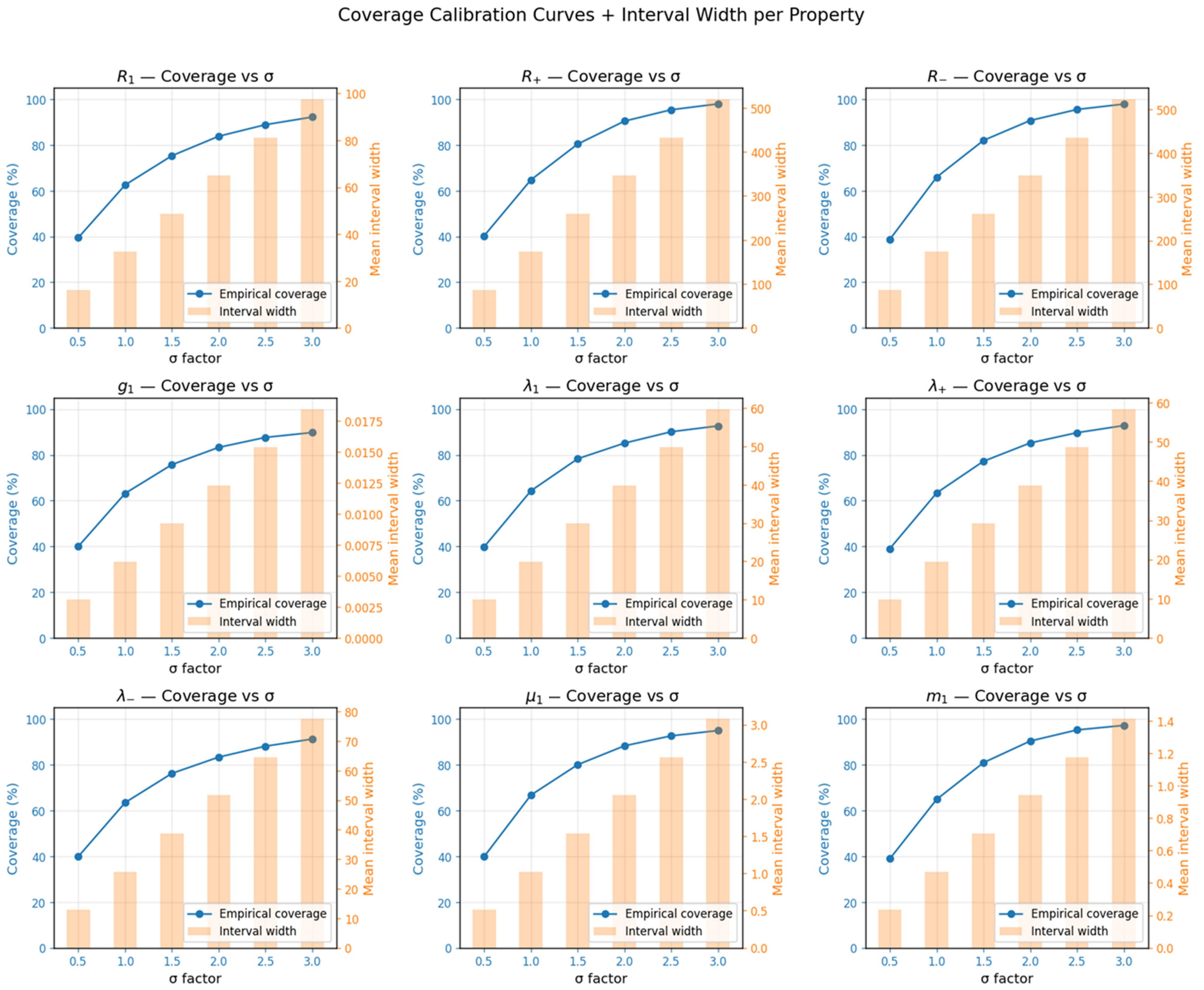

Figure S3. Calibration curves of the RFlocal model, showing coverage and mean interval width for different targets as a function of the uncertainty scaling factor ($k{\cdot}\sigma$).

4. Genetic algorithms details.

The genetic algorithm employed in this work is based on a conventional evolutionary optimization framework implemented through the **DEAP** (Distributed Evolutionary Algorithms in Python) library. In this scheme, each candidate molecule is encoded as a 16-element vector corresponding to the substitution pattern of a [6]helicene. The algorithm operates on a population-based search strategy in which individuals evolve through repeated cycles of selection, crossover, and mutation. Fitness evaluation is performed using a local surrogate model, which combines *k*-nearest neighbors with an inverse-distance-weighted Random Forest regressor (our local model described in section 2). This hybrid predictor provides locally informed estimates of the target property or properties and is used to score each individual.

At initialization, the population (typically 1000 compounds) is generated by sampling only chemically valid substituent values extracted from the dataset, and individuals are optionally constrained to preserve molecular symmetry. As a special case, the search for high g_{abs} values incorporates additional considerations to improve efficiency and coverage of promising candidates. Specifically, the initial population is increased to 5000 individuals, and 20% of the population is constrained to have a predicted g_{abs} above 0.01. In all cases, the genetic operators follow standard evolutionary practice: crossover is implemented as a two-point recombination between parent vectors, while mutation randomly alters allowed positions with a predefined probability. Selection of surviving individuals is performed through tournament selection, and elitism is enforced by retaining the top-performing candidates across generations.

A notable feature of this implementation is its flexible constraint system, designed to preserve chemical feasibility and incorporate user-defined design rules. Specifically, the algorithm allows:

1. **Prohibited substituents** – Certain substituent values can be excluded entirely from the search space.

2. **Forbidden positions** – Selected positions within the 16-element vector can be locked, preventing them from being modified or used during initialization and mutation.

3. **Fixed positions** – Positions can be preassigned to a specific substituent value; if molecular symmetry is activated, their mirror positions are automatically enforced.

4. **Position-specific allowed sets** – Individual positions may be restricted to a predefined subset of substituent values, enabling fine-grained chemical control (e.g., only electron-donating groups at a given site).

5. **Optional positional symmetry** – When activated, only half of the vector is optimized and the remaining positions are automatically mirrored, ensuring symmetric helicenes and reducing the effective search space.

These constraint mechanisms are systematically applied during individual creation, mutation, and crossover, ensuring that all generated molecules remain compliant with the specified design rules.

Overall, this custom genetic algorithm provides a robust and extensible framework capable of exploring large combinatorial chemical spaces while respecting strict structural or chemical constraints. The codes used in this study are available at https://github.com/rguceda/-6-Helicenes.

5. Optimization cases: detailed results

5.1. Maximizing R_+

Table S3. TD-DFT Values of the Proposed Candidates.

**Substituents**	$R_+ 10^{40}$**/esu cm erg G**$^{-1}$
None ([6]Helicene)	694.57
2-Br_3-Br_14-Br_15-Br	942.16
3-C≡C-Ph_5-CH_3_6-CH_3_13-C≡C-Ph_12-CH_3_11-CH_3	1467.56
3- C≡C-Ph_6-CH_3_11-CH_3_13-C≡C-Ph	1380.56
3-C≡C-Ph_8-C≡CH_9-C≡CH_13-C≡C-Ph	1360.08
3-C≡C-Ph_8-CH_3_9-CH_3_13-C≡C-Ph	1359.58
Excluding alkynes (C≡C-Ph and C≡CH)	
3-I_15-Br	844.20
2-Br_15-Br	843.81
2-Br_15-I	838.71
3-Br_15-Br	837.09
2-Br_14-Cl_15-SH	821.67
3-Br_15-I	803.20
15-Br	781.54
14-OMe_15-Br	780.35
14-I	778.87

5.2. Maximizing R_+ and R_-

Table S4. TD-DFT Values of the Proposed Candidates.

Substituents	$R_+ 10^{40}$/esu cm erg G^{-1}	$R_- 10^{40}$/esu cm erg G^{-1}
None ([6]Helicene)	694.57	–514.92
3-I	778.87	-615.05
3-Cl	713.64	-566.87
2-C≡CH_15-C≡CH	987.89	-510.48
3-C≡CH_5-F_14-Br	850.74	-690.94
3-C≡CH_14-Br	849.39	-696.50
14-SMe_15-C≡CH	818.83	-763.98
3-F_14-SMe_15-C≡CH	790.21	-711.68
3-COOH_13-F_14-CN	785.10	-727.43
3-C≡CH_13-CH_3_14-Br	763.55	-686.88
3-CN_14-COOH	753.47	-872.52

5.3. Maximizing R_+ and λ_+

Table S5. TD-DFT Values of the Proposed Candidates.

Substituents	$R_+ 10^{40}$/esu cm erg G^{-1}	λ_+/nm
None ([6]Helicene)	694.57	342.26
2-Br_14-F_15-Cl	798.47	344.52
3-CN_14-CN	859.12	353.02
3-CN_14-COOH	753.47	354.41
3-COOH_14-CN_16-OMe	706.27	356.86
2-Br_14-C≡CH_15-COOH	784.99	358.56
2-CN_3-COOH_10-CH_3_14-C≡CH_15-C≡CH_16-F	758.63	374.12
2-CN_3-COOH_10-SMe_14-C≡CH_15C≡C-Ph	898.17	391.23

5.4. Maximizing R_- and λ_-

Table S6. TD-DFT Values of the Proposed Candidates.

Substituents	$R_- 10^{40}$/esu cm erg G^{-1}	λ_-/nm
None ([6]Helicene)	–514.92	254.72
1-F_2-SMe_3-COOH_5-F_8-Cl_14-CN	-670.68	264.38
3-CHO_9-COOH_11-COOH_14-CHO	-707.58	284.28
3-SH_8-F_13-CH_3_14-COOH	-646.96	275.90
1-F_2-SMe_3-COOH_14-CN	-574.28	262.15
3-SH_13-CH_3_14-COOH	-685.36	276.08
5-F_14-SH	-780.12	267.99
5-SMe_14-SH	-749.24	267.81
3-SH	-739.28	266.80

5.5. Maximizing m_1

Table S7. TD-DFT Values of the Proposed Candidates.

**Substituents**	**$m_1 10^{20}$/erg G^{-1}**
None ([6]Helicene)	0.36
3-C≡C-Ph_12-C≡C-Ph	3.51
5-C≡C-Ph_13-C≡CH_14-C≡C-Ph	3.29
2-C≡C-Ph_4-CH_3_10-C≡C-Ph_14-C≡CH	3.17
5-C≡C-Ph_9-C≡CH_14-C≡C-Ph	3.17
2-C≡CH_3-CH_3_7-C≡C-Ph_15-C≡C-Ph	3.08
1-CH_3_2-C≡C-Ph_4-C≡CH_10-C≡CH_14-C≡C-Ph	2.95
1-I_2-C≡C-Ph_5-C≡C-Ph_15-I	2.62
2-Br_5-C≡C-Ph_14-C≡CH_15-OMe	2.55
2-SH_3-OMe_7-Cl_12-SH_15-C≡C-Ph	2.29
2-C≡C-Ph_3-OMe_7-Cl_12-SH_15-C≡C-Ph	2.23
3-C≡C-Ph_5-SMe_12-C≡C-Ph_14-SH	2.22
2-SH_3-OMe_7-Cl_15-C≡C-Ph	2.13
2-SH_3-OMe_5-C≡CH_7-Cl_13-C≡CH_15-C≡C-Ph	2.12
1-CHO_3-Br_4-Cl_11-COOH_13-CH_3_14-C≡C-Ph_15-OH_16-Cl	2.04
1-CHO_2-NH_2_3-C≡C-Ph_4-SMe_8-SH_11-OMe_15-OH	1.97
1-CHO_2-NH_2_3-C≡C-Ph_4-F_7-C≡C-Ph_15-OH	1.97

5.6. Designing λ_1

5.6.1. λ_1 ~ 400 nm

Table S8. TD-DFT Values of the Proposed Candidates.

Substituents	λ_1/nm
9-I_10-C≡C-Ph	399.86
4-CN_14-OMe_16-OMe	400.20
3-CHO_10-CN	399.67
7-C≡CH_11-CN	400.55
3-OH_6-F_9-OH_10-OMe_16-OMe	399.44
8-C≡CH_13-CN_15-Cl	399.30
1-CH_3_13-SH_16-SH	398.25
3-OH_6-F_8-C≡CH_16-OMe	396.80
8-C≡CH_12-Cl_13-COOH_15-Cl	395.20
10-CN_16-Br	405.01
9-SH_12-Cl_13-COOH_15-Cl	394.60
1-SH_7-SH_11-CN	406.19
3-CHO_12-SH	406.22
7-SH_11-CN	393.53
3-OH_6-F_8-C≡C-Ph	393.01
1-SH_3-OH_6-F_8-C≡C-Ph	408.11
1-OH_5-C≡CH_8-CH3_11-SMe	408.14
1-CH_3_5-OH_16-SH	408.20
8-C≡CH_12-I	390.57
3-COOH_8-I	390.02

5.6.2. λ_1 ~ 450 nm

Table S9. TD-DFT Values of the Proposed Candidates.

Substituents	λ_1/nm
1-CH_3_3-CHO_4-OMe_5-C≡C-Ph_16-SH	441.38
1-OH_2-NO_2_4-F_6-NH_2_9-OMe_12-OMe_15-C≡CH _16-Cl	445.13
1-NO_2_6-I_9-COOH_12-I_14-C≡CH_16-COOH	445.14
2-SH_3-C≡CH_8-Br_9-NH_2_14-C≡CH_15-C≡CH	451.39
7-CHO_8-OMe_11-OH_12-CHO_13-CH_3	452.47
1-CN_3-C≡CH_7-CHO_12-NO_2_14-Cl_15-OMe	453.16
1-NH2_2-OMe_7-I_8-Cl_9-CH_3	456.73
2-COOH_3-COOH_6-SH_7-CN_9-SH_10-SMe_12-CH_3_14-CHO_15-NO_2	457.87
2-SH_3-SH_10-F_11-CHO_12-CN_13-F	459.26
1-CN_5-CN_6-CN_10-OMe_15-C≡CH_16-F	461.43

5.6.3. λ_1 ~ 500 nm

Table S10. TD-DFT Values of the Proposed Candidates.

Substituents	λ_1/nm
2-I_5-NH_2_7-CN_8-NH_2_9-OH_11-CN_14-I	493.07
1-Cl_3-CHO_5-CH_3_6-Cl_8-NO_2_9-OH_10-NO_2_11-Cl_12-COOH_14-Br_15-I	494.97
1-NO_2_5-OMe_7-Br_9-NO_2_10-NO_2_12-NO_2_14-C≡CH_15-OMe_16-NO_2	497.60
2-CN_3-COOH_5-NH_2_16-NO_2	500.30
1-NO_2_3-NO_2	500.66
2-NO_2_5-CN_6-CN_8-F_10-SH	501.80
3-Br_4-C≡CH_7-CHO_11-NO_2_12-NO_2_14-C≡CH_16-CN	502.87
4-C≡CH_7-CH_3_11-NO_2_12-NO_2_14-C≡CH_16-CN	503.47
3-Br_7-CHO_11-NO_2_12-NO_2_14-C≡CH_16-CN	503.54
3-CH_3_4-SH_5-Cl_7-OMe_12-OH_16-NO_2	505.83
2-CN_3-COOH_5-NH_2_7-C≡CH_8-C≡CH_11-SMe_12-OH_16-NO_2	506.48
1-OH_2-OH_4-OMe_6-NO_2_9-F_10-SH_12-COOH_14-OMe_15-NO_2	506.87
4-C≡CH_8-OMe_11-NO_2_14-C≡CH_16-NO_2	507.88
4-C≡CH_8-OMe_11-COOH_12-SH_14-C≡CH_16-NO_2	509.43
2-I_5-NH_2_7-CN_8-NH_2_9-OH_11-CN_14-I	493.07

5.6.4. λ_1 ~ 550 nm

Table S11. TD-DFT Values of the Proposed Candidates.

Substituents	λ_1/nm
3-SH_7-OMe_8-C≡CH_10-SMe_12-NO_2_14-CH_3_15-NO_2_16-NO_2	551.84
2-I_4-NH_2_5-CH_3_9-OH_11-C≡C-Ph_13-C≡CH_15-I_16-NO_2	562.64
2-NH_2_3-CN_4-NO_2_5-I_7-CH_3_9-COOH_10-I_12-NO_2_16-NH_2	543.45
2-OH_3-Cl_4-C≡CH_5-NH_2_6-F_7-CH_3_9-OMe_10-C≡C-Ph_12-OMe_15-OMe_16-NO_2	547.97
1-NO_2_2-NO_2_4-CHO_5-I_6-NH_2_7-C≡CH_8-NH_2_10-SH_12-C≡CH_14-CHO_15-SH_16-NH_2	564.72

5.6.5. λ_1 ~ 600 nm

Table S12. TD-DFT Values of the Proposed Candidates.

Substituents	λ_1/nm
4-SMe_7-NH2_11-I_12-I_14-NO_2_16-NO_2	588.72
2-NO_2_3-NO_2_4-OMe_5-I_10-OMe_13-CN_15-CH_3_16-SMe	598.79
2-NO_2_3-NO_2_5-C≡CH_7-CHO_9-OH_10-OH_11-NH_2_13-C≡CH_14-I	595.69
2-SMe_9-CH_3_11-CN_14-NO_2_15-NO_2_16-Cl	591.20
1-NO_2_3-NO_2_4-C≡CH_8-SH_10-OH_12-SMe_13-SH_14-CH_3	621.75

5.7. Exceptional g_{abs} values for the less energetic transition in *P*-[6]helicenes

Table S13. TD-DFT Values of the Proposed Candidates.

Substituents	$\mu_1 10^{18}$/esu cm	$m_1 10^{20}$/erg G^{-1}	g_1
1-OH_3-SH_6-SH	0.02	0.25	0.389
4-OH_10-F_13-Br_16-NO_2	0.21	0.77	0.144
1-F_3-F_9-F_14-Cl_15-Cl_16-Br	0.07	0.29	0.141
1-NO_2_2-NO_2_7-CN	0.20	0.62	0.114
1-F_7-SH_8-CHO_11-NO_2_16-NO_2	0.11	0.41	0.113
1-F_2-F_3-Br_4-I_5-I_6-F	0.09	0.32	0.112
1-NO_2_6-NO_2_9-COOH	0.40	1.11	0.108
6-CN_1-NO_2_5-COOH_10-COOH	0.35	1.00	0.107
1-OH_13-CHO_14-NO_2_15-OMe	0.34	1.31	0.105
1-NO_2_6-NO_2_9-CN	0.43	1.11	0.102

As can be observed, the driving force behind these exceptionally high dissymmetry factors is the remarkably small value of the electric transition dipole moment, which is one to two orders of magnitude lower than typically encountered.

It should be noted that these compounds may possess thermally accessible conformers in which the electric dipole moment is higher, leading to a corresponding reduction in the dissymmetry factor *g*. For this reason, the conformers corresponding to the highest g_{abs} values were explicitly generated using the CREST software,[10] and Boltzmann-weighted values were subsequently computed (Table S14). These calculations confirm that, at least in general terms, the outstanding dissymmetry factors are largely preserved when accessible conformers are taken into account.

Table S14. Boltzmann-weighted dissymmetry factors and transition dipole moments for the compounds with highest g_{abs}

Substituents	Conformer	Population/%	$\mu_1 10^{18}$/esu cm	$m_1 10^{20}$/erg G^{-1}	g_1
1-OH_3-SH_6-SH	1	41.88	0.02	0.25	3.89×10^{-1}
	2	40.39	0.12	0.21	-1.69×10^{-2}
	3	9.98	0.35	0.15	-3.74×10^{-4}
	4	7.72	0.28	0.18	1.47×10^{-3}
Boltzmann-weighted values			0.11	0.22	0.156
1-F_3-F_9-F_14-Cl_15-Cl_16-Br	1	100	0.07	0.29	0.141
Boltzmann-weighted values			0.07	0.29	0.141
4-OH_10-F_13-Br_16-NO_2	1	0.95	0.21	0.78	1.09×10^{-1}*
	2	0.05	0.21	0.78	1.44×10^{-1}*
Boltzmann-weighted values			0.21	0.78	0.104
1-NO_2_2-NO_2_7-CN	1		0.2	0.62	0.114
Boltzmann-weighted values			0.2	0.62	0.114

* They exhibit different g_{abs} values, as they differ only slightly in their electric ($2.123\cdot10^{-19}$ esu cm for 1 and $2.079\cdot10^{-19}$ esu cm for 2) and magnetic ($7.815\cdot10^{-21}$ erg G^{-1} for 1 and $7.751\cdot10^{-21}$ erg G^{-1} for 2) dipole transition moments.

5.8. Exceptional CPL emitters in (*P*)-[6]helicenes

Table S15. TD-DFT Values of the Proposed Candidates.

Substituents	$S_0 \rightarrow S_1$			$S_1 \rightarrow S_0$			
	$\mu_1 10^{18}$/esu cm	$m_1 10^{20}$/erg G^{-1}	g_1	$\mu_1 10^{18}$/esu cm	$m_1 10^{20}$/erg G^{-1}	g_1	*f*
1-NO_2_16-CN	0.93	1.35	5.08×10^{-2}	1.12	1.42	3.80×10^{-2}	0.0086
1-NO_2_4-OH	0.87	1.23	5.05×10^{-2}	0.92	1.26	4.88×10^{-2}	0.0062
1-NO_2_13-CHO_14-CN	1.14	1.04	2.83×10^{-2}	1.11	1.43	3.83×10^{-2}	0.0089
1-NO_2_4-OH_12-F	0.84	1.26	5.60×10^{-2}	0.53	0.57	$-7.75E\times10^{-3}$	0.0012
1-NO_2_5-Cl_14-CN	0.85	1.03	4.32×10^{-2}	1.15	0.71	1.69×10^{-2}	0.0100
1-NO_2_9-F_16-CN	0.87	1.29	5.46×10^{-2}	1.14	1.42	3.72×10^{-2}	0.0092
1-CHO_2-Br_15-OH_16-NO_2	0.77	1.51	5.68×10^{-2}	0.41	1.85	1.10×10^{-2}	0.0004
1-NO_2_12-I_13-CN_16-COOH	0.82	1.32	5.15×10^{-2}	1.08	1.52	3.86×10^{-2}	0.0082
1-NO_2_6-C≡CH_10-NO2_14-CN	1.51	1.12	1.80×10^{-2}	1.08	1.44	4.83×10^{-2}	0.0086
1-CHO_5-OMe_8-C≡CH_14-F_16-NO_2	0.92	1.22	4.34×10^{-2}	0.42	1.12	9.94×10^{-2}	0.0008
1-NO_2_3-C≡CH_7-OH_11-OMe_16-NO_2	1.06	0.94	1.15×10^{-2}	1.13	1.11	1.24×10^{-2}	0.0071
1-NO_2_4-CH_3_12-C≡CH_14-Br_16-NO_2	1.13	1.29	3.36×10^{-2}	1.50	0.41	6.86×10^{-3}	0.0159
1-NO_2_5-OMe_7-CH_3_10-C≡CH_14-Br_16-COOH	0.91	0.80	3.09×10^{-2}	1.09	0.44	1.13×10^{-2}	0.0073
1-NO_2_13-SMe_14-COOH	0.76	1.18	5.50×10^{-2}	1.00	1.38	4.21×10^{-2}	0.0069
1-NO_2_5-SH_7-COOH_16-NO_2	0.79	1.18	5.10×10^{-2}	0.73	1.15	5.41×10^{-2}	0.0033
1-NO_2_3-F_13-CHO_15-C≡CH	0.73	1.16	5.04×10^{-2}	0.88	1.32	4.74×10^{-2}	0.0050
1-NO_2_5-SH_6-COOH_11-CN_14-COOH	0.85	0.95	4.21×10^{-2}	1.43	0.39	5.08×10^{-3}	0.0147
1-NO_2_3-NH_2_6-Br_11-Br_16-NO_2	1.21	1.18	2.54×10^{-2}	1.22	0.55	1.47×10^{-2}	0.0091

Table S16. Relevant parameters for [6]helicenes bearing one NO_2 group.

NO_2 position	$\mu_1 10^{18}$/esu cm	$m_1 10^{20}$/erg G^{-1}	cos θ	g_1
1	0.61	1.06	0.88	6.14×10^{-2}
2	0.66	0.42	0.10	2.43×10^{-3}
3	2.68	0.95	0.32	4.60×10^{-3}
4	0.90	0.20	-0.26	-2.30×10^{-3}
5	1.61	0.53	-0.20	-2.59×10^{-3}
6	1.74	0.58	0.15	2.02×10^{-3}
7	2.19	0.54	0.13	1.32×10^{-3}
8	2.60	0.22	-0.76	-2.59×10^{-3}

Table S17. Relevant parameters for symmetrical [6]helicenes bearing two NO_2 groups.

NO_2 position	$\mu_1 10^{18}$/esu cm	$m_1 10^{20}$/erg G^{-1}	cos θ	g_1
1,16	0.97	1.54	0.91	5.77×10^{-2}
2,15	1.28	0.15	-1	-4.53×10^{-3}
3,14	1.46	0.13	0.88	3.27×10^{-3}
4,13	2.47	1.37	0.18	4.14×10^{-3}
5,12	2.01	0.67	-0.05	-7.12×10^{-4}
6,11	1.99	0.91	0.28	5.20×10^{-3}
7,10	2.50	0.79	0.33	4.24×10^{-3}
8,9	2.65	0.43	-0.12	-8.29×10^{-4}

Table S18. TD-DFT Values of the Proposed Candidate excluding NO_2 group

Substituents	$S_0 \rightarrow S_1$			$S_1 \rightarrow S_0$			
	$\mu_1 10^{18}$/esu cm	$m_1 10^{20}$/erg G^{-1}	g_1	$\mu_1 10^{18}$/esu cm	$m_1 10^{20}$/erg G^{-1}	g_1	f
1-CHO_15-CHO	1.50	1.67	3.90×10^{-2}	2.67	2.27	3.40×10^{-2}	0.0635
2-CHO_10-Cl_16-CN	1.64	1.22	1.26×10^{-2}	3.96	2.39	8.01×10^{-3}	0.1452
1-COOH_4-CN_15-Cl	1.76	1.35	1.04×10^{-2}	1.56	1.63	2.49×10^{-2}	0.0197
2-COOH_7-C≡CH_15-NH2	1.16	0.76	1.23×10^{-2}	5.10	2.07	3.45×10^{-3}	0.2306
4-SMe_15-I_16-CN	1.27	1.17	1.52×10^{-2}	2.31	1.55	9.08×10^{-3}	0.0468
2-CHO_10-CN_15-CN_16-CN	0.99	0.85	2.23×10^{-2}	2.84	1.28	4.77×10^{-3}	0.0760
2-C≡CH_10-CN_13-COOH_14-NH2_16-CN	1.11	1.00	3.58×10^{-2}	3.47	2.33	1.10×10^{-2}	0.1078
2-CHO_4-CN_10-CN_15-CN_16-CN	0.90	0.78	1.61×10^{-2}	1.96	1.59	1.33×10^{-2}	0.0381
1-OMe_6-F_10-CN_15-CN_16-CN	1.06	0.78	1.81×10^{-2}	0.81	0.25	-1.13×10^{-3}	0.0051
1-CN_2-C≡CH_6-C≡CH_10-CN_13-CN_16-CN	1.57	1.49	1.69×10^{-2}	2.79	2.17	1.70×10^{-2}	0.0695
1-COOH_2-COOH_4-SMe_14-SH_15-I_16-CN	1.53	1.69	2.40×10^{-2}	2.84	0.76	-4.17×10^{-3}	0.0616

5.9. Exceptional CPL emitters in (*P*)-[6]helicenes: what about their synthesis?

Table S19. TD-DFT Values of the Proposed Candidates.

Substituents	$S_0 \rightarrow S_1$			$S_1 \rightarrow S_0$			
	$\mu_1 10^{18}$/esu cm	$m_1 10^{20}$/erg G^{-1}	g_1	$\mu_1 10^{18}$/esu cm	$m_1 10^{20}$/erg G^{-1}	g_1	f
1-NO_2_3-COOH_7-I_10-I_14-COOH_16-NO_2	0.82	1.29	6.13×10^{-2}	1.24	1.33	2.59×10^{-2}	0.0101
1-NO_2_7-OMe_8-F_9-F_10-OMe_16-NO_2	0.94	1.49	6.08×10^{-2}	1.23	1.20	2.23×10^{-2}	0.0087
1-NO_2_3-COOH_6-Br_8-F_9-F_11-Br_14-COOH_16-NO_2	1.08	1.58	5.72×10^{-2}	1.61	1.59	2.56×10^{-2}	0.0194
1-NO_2_3-F_5-F_7-CHO_10-CHO_12-F_14-F_16NO_2	0.92	1.43	5.60×10^{-2}	0.87	1.42	6.28×10^{-2}	0.0051
1-NO_2_3-F_7-CHO_10-CHO_14-F_16-NO_2	0.99	1.52	5.43×10^{-2}	1.10	1.54	5.02×10^{-2}	0.0087
1-NO2_2-Br_3-I_8-F_9-F_14-I_15-Br_16-NO2	1.07	1.38	5.08×10^{-2}	1.25	0.38	-1.21×10^{-2}	0.0138
1-NO_2_2-Br_3-I_14-I_15-Br_16-NO_2	1.00	1.28	5.02×10^{-2}	1.34	1.42	4.04×10^{-2}	0.0148
1-NO_2_3-SH_14-SH_16-NO_2	1.16	1.41	4.78×10^{-2}	1.87	1.36	1.54×10^{-2}	0.0229
1-NO_2_2-Br_8-I_9-I_15-Br_16-NO_2	0.95	1.13	4.47×10^{-2}	1.45	1.40	3.57×10^{-2}	0.0171
1-NO_2_3-Br_5-OH_8-I_9-I_12-OH_14-Br_16-NO_2	1.02	1.22	4.25×10^{-2}	1.57	1.18	2.38×10^{-2}	0.0171
1-NO_2_3-F_4-Br_7-I_10-I_13-Br_14-F_16-NO_2	0.94	1.38	3.94×10^{-2}	1.58	1.30	2.08×10^{-2}	0.0201
1-NO_2_2-Cl_4-Br_7-NO_2_8-CH_3_9-CH_3_10-NO_2_13-Br_15-Cl_16-NO_2	0.94	1.22	3.87×10^{-2}	1.14	1.41	3.46×10^{-2}	0.0095
1-NO_2_5-C≡CH_6-Br_7-CHO_10-CHO_11-Br_12-C≡CH_16-NO_2	1.07	1.48	3.81×10^{-2}	1.42	1.49	2.64×10^{-2}	0.0141
1-NO_2_2-COOH_3-NH2_7-NO2_10-NO2_14-NH2_15-COOH_16-NO_2	1.24	1.35	3.79×10^{-2}	0.74	0.26	-5.52×10^{-3}	0.0012
1-NO_2_2-F_4-SH_8-CH_3_9-CH_3_13-SH_15-F_16-NO_2	1.17	1.49	3.56×10^{-2}	1.13	1.39	2.44×10^{-2}	0.0082
1-NO_2_3-F_4-I_13-I_14-F_16-NO_2	1.27	1.23	2.63×10^{-2}	0.88	0.18	-8.17×10^{-3}	0.0058
1-NO_2_4-SH_7-CH_3_10-CH_3_13-SH_16-NO_2	1.48	1.34	1.79×10^{-2}	0.78	1.18	4.45×10^{-2}	0.0038
1-NO2_4-NH2_5-F_12-F_13-NH2_16-NO2	2.01	1.38	1.67×10^{-2}	2.62	1.31	1.07×10^{-2}	0.0573
1-NO_2_4-SH_7-F_10-F_13-SH_16-NO_2	1.54	1.27	1.59×10^{-2}	0.81	1.21	4.67×10^{-2}	0.0041

Table S20. TD-DFT Values of the Proposed Candidates excluding NO_2 group.

Substituents	$S_0 \rightarrow S_1$			$S_1 \rightarrow S_0$			
	$\mu_1 10^{18}$/esu cm	$m_1 10^{20}$/erg G^{-1}	g_1	$\mu_1 10^{18}$/esu cm	$m_1 10^{20}$/erg G^{-1}	g_1	f
1-CHO_4-F_13-F_16-CHO	1.26	1.82	5.20×10^{-2}	2.51	2.14	1.99×10^{-2}	0.0563
1-CHO_5-F_12-F_16-CHO	1.28	1.67	4.61×10^{-2}	2.22	1.96	2.26×10^{-2}	0.0425
1-CHO_2-I_15-I_16-CHO	1.32	1.91	3.48×10^{-2}	2.08	1.99	2.37×10^{-2}	0.0347
2-COOH_4-OH_6-F_11-F_13-OH_15-COOH	0.83	0.93	3.29×10^{-2}	3.74	2.35	7.39×10^{-3}	0.1339
2-CN_5-F_12-F_15-CN	0.76	0.92	3.18×10^{-2}	2.39	2.00	1.76×10^{-2}	0.0616
2-CN_6-SMe_11-SMe_15-CN	0.88	0.69	2.66×10^{-2}	3.85	1.84	5.51×10^{-3}	0.1462
1-F_2-CN_15-CN_16-F	0.93	0.80	2.49×10^{-2}	2.43	1.82	1.55×10^{-2}	0.0630
2-CN_6-F_11-F_15-CN	1.11	0.90	1.99×10^{-2}	2.29	2.76	1.29×10^{-2}	0.0582
2-COOH_7-F_10-F_15-COOH	1.43	0.96	1.14×10^{-2}	3.90	2.38	7.88×10^{-3}	0.1513
1-CHO_4-SMe_13-SMe_16-CHO	1.56	1.40	1.12×10^{-2}	1.20	1.61	3.43×10^{-2}	0.0126

Table S21. TD-DFT Values of the Proposed Candidates excluding NO_2 group and position 1.

Substituents	$S_0 \rightarrow S_1$			$S_1 \rightarrow S_0$			
	$\mu_1 10^{18}$/esu cm	$m_1 10^{20}$/erg G^{-1}	g_1	$\mu_1 10^{18}$/esu cm	$m_1 10^{20}$/erg G^{-1}	g_1	f
2-C≡CH_3-F_14-F_15-C≡CH	1.02	1.01	3.80×10^{-2}	2.20	2.13	2.89×10^{-2}	0.0525
4-NH_2_6-SMe_11-SMe_13-NH_2	0.87	0.80	3.66×10^{-2}	3.96	1.53	3.87×10^{-2}	0.1440
2-C≡C-Ph_3-SMe_14-SMe_15-C≡C-Ph	2.50	1.98	2.85×10^{-2}	2.61	2.58	3.59×10^{-2}	0.0643
2-C≡CH_15-C≡CH	1.03	0.98	2.78×10^{-2}	2.35	1.96	2.03×10^{-2}	0.0609
2-C≡CH_5-F_12-F_15-C≡CH	0.95	0.99	2.58×10^{-2}	2.31	1.83	1.78×10^{-2}	0.0586
2-C≡C-Ph_3-F_14-F_15-C≡C-Ph	3.53	2.21	2.47×10^{-2}	3.67	2.62	2.66×10^{-2}	0.1329
2-C≡CH_3-SH_14-SH_15-C≡CH	0.81	0.45	2.08×10^{-2}	3.10	2.53	1.64×10^{-2}	0.0925
2-C≡C-Ph_3-F_6-SMe_11-SMe_14-F_15-C≡C-Ph	2.93	1.50	1.91×10^{-2}	3.06	1.98	2.17×10^{-2}	0.0882

5.10. Opening the black boxes: from AI predictions to molecular design

Table S22. TD-DFT Values of the Proposed Candidates.

Substituents	$S_0 \rightarrow S_1$			$S_1 \rightarrow S_0$			
	$\mu_1 10^{18}$/esu cm	$m_1 10^{20}$/erg G^{-1}	g_1	$\mu_1 10^{18}$/esu cm	$m_1 10^{20}$/erg G^{-1}	g_1	f
1-CN_2-Br_15-Br_16-CN	1.76	1.81	1.44×10^{-2}	1.84	0.39	8.38×10^{-3}	0.0362
1-CN_2-C≡CH_15-C≡CH_16-CN	1.71	2.02	3.38×10^{-2}	2.16	2.37	2.47×10^{-2}	0.0452
1-CN_2-Cl_15-Cl_16-CN	1.82	1.67	1.00×10^{-2}	2.72	2.19	6.68×10^{-3}	0.0752
1-CN_2-C≡C-Ph_15-C≡C-Ph_16-CN	2.95	2.53	3.38×10^{-2}	2.83	2.66	3.62×10^{-2}	0.0735
1-NO_2_2-Br_15-Br_16-NO_2	0.94	1.35	5.18×10^{-2}	1.31	1.49	3.97×10^{-2}	0.0143
1-NO_2_2-C≡CH_15-C≡CH_16-NO_2	0.85	1.30	4.98×10^{-2}	1.23	0.18	-5.94×10^{-3}	0.0123
1-NO_2_2-Cl_15-Cl_16-NO_2	0.92	1.31	5.15×10^{-2}	1.25	1.52	4.32×10^{-2}	0.0131
1-NO_2_2-C≡C-Ph_15-C≡C-Ph_16-NO_2	2.28	0.32	-5.63×10^{-3}	2.63	0.29	-4.39×10^{-3}	0.0568

6. References.

[1] C. Hansch, A. Leo, R. W. Taft, *Chem. Rev.* **1991**, *91*, 165–195.

[2] M. J. Frisch, G. W. Trucks, H. B. Schlegel, G. E. Scuseria, M. A. Robb, J. R. Cheeseman, G. Scalmani, V. Barone, B. Mennucci, G. A. Petersson, H. Nakatsuji, M. Caricato, X. Li, H. P. Hratchian, A. F. Izmaylov, J. Bloino, G. Zheng, J. L. Sonnenberg, M. Hada, M. Ehara, K. Toyota, R. Fukuda, J. Hasegawa, M. Ishida, T. Nakajima, Y. Honda, O. Kitao, H. Nakai, T. Vreven, J. A. Montgomery Jr., J. E. Peralta, F. Ogliaro, M. Bearpark, J. J. Heyd, E. Brothers, K. N. Kudin, V. N. Staroverov, R. Kobayashi, J. Normand, K. Raghavachari, A. Rendell, J. C. Burant, S. S. Iyengar, J. Tomasi, M. Cossi, N. Rega, J. M. Millam, M. Klene, J. E. Knox, J. B. Cross, V. Bakken, C. Adamo, J. Jaramillo, R. Gomperts, R. E. Stratmann, O. Yazyev, A. J. Austin, R. Cammi, C. Pomelli, J. W. Ochterski, R. L. Martin, K. Morokuma, V. G. Zakrzewski, G. A. Voth, P. Salvador, J. J. Dannenberg, S. Dapprich, A. D. Daniels, O. Farkas, J. B. Foresman, J. V. Ortiz, J. Cioslowski, D. J. Fox, Gaussian09. Revission B.01., **2010**.

[3] Y. Zhao, D. G. Truhlar, *Theor. Chem. Acc.* **2008**, *120*, 215–241.

[4] A. Schäfer, H. Horn, R. Ahlrichs, *J. Chem. Phys.* **1992**, *97*, 2571–2577

[5] Bergner, M. Dolg, W. Küchle, H. Stoll, H. Preuß, *Mol. Phys.* **1993**, *80*, 1431–1441.

[6] S. Miertuš, E. Scrocco, J. Tomasi, *Chem. Phys.* **1981**, *55*, 117–129.

[7] R. G. Uceda, A. Gijon, S. Míguez-Lago, C. M. Cruz, V. Blanco, F. Fernández-Álvarez, L. Álvarez de Cienfuegos, M. Molina-Solana, J. Gómez-Romero, D. Miguel, A. J. Mota, J. M. Cuerva, *Angew. Chem. Int. Ed.* **2024**, *63*, e202409998.

[8] A. D. Becke, *J. Chem. Phys.* **1993**, *98*, 5648–5652.

[9] C. Adamo, M. Cossi, V. Barone, *J. Mol. Struct.: THEOCHEM.*, **1999**, *493*, 145-157.

[10] P. Pracht, F. Bohle, S. Grimme, *Phys. Chem. Chem. Phys.*, **2020**, *22*, 7169-7192.
